# Epigenetic priming of mammalian embryonic enhancer elements coordinates developmental gene networks

**DOI:** 10.1186/s13059-025-03658-8

**Published:** 2025-07-18

**Authors:** Christopher D. Todd, Jannat Ijaz, Fereshteh Torabi, Oleksandr Dovgusha, Stephen Bevan, Olivia Cracknell, Tim Lohoff, Stephen Clark, Ricard Argelaguet, Juliette Pearce, Ioannis Kafetzopoulos, Alice Santambrogio, Jennifer Nichols, Ferdinand von Meyenn, Ufuk Günesdogan, Stefan Schoenfelder, Wolf Reik

**Affiliations:** 1 Epigenetics Programme, Babraham Institute, Babraham Research Campus, Cambridge, CB22 3AT UK; 2 Göttingen Center for Molecular Biology, Department of Developmental Biology, University of Göttingen, Justus-Von-Liebig Weg 11, Göttingen, 37077 Germany; 3 Wellcome-MRC Cambridge Stem Cell Institute, Jeffrey Cheah Biomedical Centre, University of Cambridge, Puddicombe Way, Cambridge, CB2 0AW UK; 4 Present Address: Altos Labs, Cambridge Institute of Science, Granta Park, Cambridge, CB21 6GQ UK; 5 Present Address: Wellcome Sanger Institute, Wellcome Genome Campus, Hinxton, Cambridge, CB10 1SA UK; 6 Present Address: GSK, Gunnels Wood Road, Stevenage, SG1 2NY UK; 7 Present Address: Forbion, Gaertnerplatz 6, Munich, Germany; 8 Present Address: Institute for Cell and Tumor Biology, RWTH Aachen University Hospital, Aachen, Germany; 9 Present Address: Institute of Genetics and Cancer, The University of Edinburgh, Western General Hospital, Crewe Road, Edinburgh, EH4 2XU UK; 10 Present Address: Laboratory of Nutrition and Metabolic Epigenetics, Department of Health Sciences and Technology, ETH Zurich, Zurich, Switzerland; 11 Department of Medical and Molecular Genetics, Kings College London, London, UK

**Keywords:** Developmental biology, Enhancers, Epigenetics, Embryogenesis, Cell fate, Multi-omics

## Abstract

**Background:**

Embryonic development requires the accurate spatiotemporal execution of cell lineage-specific gene expression programs, which are controlled by transcriptional enhancers. Developmental enhancers adopt a primed chromatin state prior to their activation. How this primed enhancer state is established and maintained and how it affects the regulation of developmental gene networks remains poorly understood.

**Results:**

Here, we use comparative multi-omic analyses of human and mouse early embryonic development to identify subsets of postgastrulation lineage-specific enhancers which are epigenetically primed ahead of their activation, marked by the histone modification H3K4me1 within the epiblast. We show that epigenetic priming occurs at lineage-specific enhancers for all three germ layers and that epigenetic priming of enhancers confers lineage-specific regulation of key developmental gene networks. Surprisingly in some cases, lineage-specific enhancers are epigenetically marked already in the zygote, weeks before their activation during lineage specification. Moreover, we outline a generalizable strategy to use naturally occurring human genetic variation to delineate important sequence determinants of primed enhancer function.

**Conclusions:**

Our findings identify an evolutionarily conserved program of enhancer priming and begin to dissect the temporal dynamics and mechanisms of its establishment and maintenance during early mammalian development.

**Supplementary Information:**

The online version contains supplementary material available at https://doi.org/10.1186/s13059-025-03658-8.

## Background

Embryogenesis requires the generation of a complex array of different cell types with distinct transcriptional programs. Enhancers, cis-acting transcriptional regulatory elements [1], play an important role in the programmed establishment and maintenance of cell fates through binding of sequence-specific transcription factors (TFs) and regulating transcription of their target genes in a cell-type-specific manner [2]. Previous studies have utilized genetic analysis within disease models to highlight the potential functional importance of enhancer elements, such as the zone of polarizing activity regulatory sequence (ZRS) enhancer for the Shh gene [3]. Genetic editing and deletion of this enhancer element, resulting in polydactyly and syndactyly, has provided conclusive evidence of this enhancer’s importance in establishing the correct spatiotemporal regulation of developmental tissues [4]. While whole-genome sequencing has facilitated the investigation of genetic factors such as TF-binding motif sequences in enhancer activity, large-scale profiling of epigenetic marks has started to reveal the role of specific chromatin modifications in regulating enhancer activity. For example, active enhancers promoting transcription of associated genes are associated with the presence of the histone mark H3K27ac [5–8].

Studies of developmental programs have shown that lineage-specific enhancers often transition through preactive states ahead of their subsequent activation; this “primed” enhancer state is defined as enhancers marked with the neutral enhancer-associated histone modification H3K4me1 in the absence of the active enhancer mark H3K27ac [9, 10]. A distinct “poised” preactive state is defined as enhancers marked with primed (H3K4me1) and repressive (H3K27me3) marks [7, 9]. These preactive enhancer states are hypothesized to prepare the enhancer region for activation upon binding of lineage-specific TFs to facilitate cell-fate-associated changes of transcriptional programs. This is seen in various developmental processes including *Drosophila* mesoderm formation, zebrafish embryogenesis, neural crest development, cardiac lineage formation, and lymphogenesis [11–15].

Recent functional genomics and multi-omics approaches have begun to unravel the epigenetic regulation of enhancer activity and its role in early mammalian development. For example, tracing the epigenetic dynamics of lineage-specific enhancer elements during mouse gastrulation has revealed priming by DNA hypomethylation and accessible chromatin at neuroectodermal enhancers, but not mesodermal or endodermal enhancers, within the epiblast [16]. However, several pertinent questions remain surrounding enhancer priming and its role in mammalian embryonic development. These include the following: (i) How is enhancer priming established and maintained? (ii) How do lineage-specific enhancers undergo priming during human embryonic development? (iii) What are the temporal dynamics of enhancer priming? In this study, we address these questions through multi-omic analysis of in vitro and in vivo models of human and mouse early embryonic development to determine the dynamics of enhancer epigenetic priming, to investigate its role in regulating cell fate decisions, and to identify potential trans-acting factors that establish the primed state at key human developmental enhancers.

## Results

### Epigenetic priming of lineage-specific enhancers in human and mouse epiblast

**Fig. 1:**
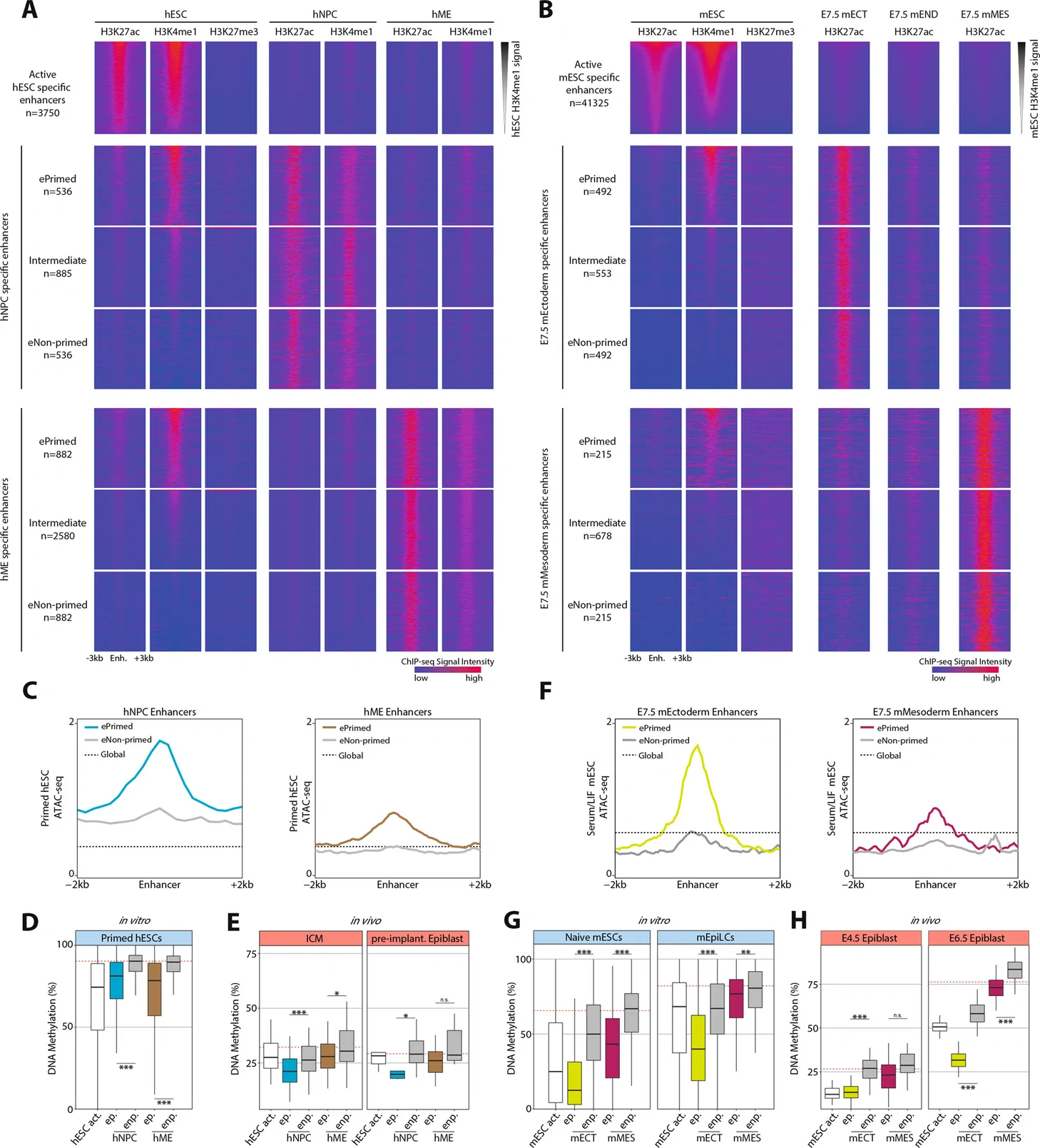
**A** Heatmaps of ChIP-seq H3K27ac, H3K4me1, and H3K27me3 data within hESC, hNPC, and hME cells in culture at enhancer elements with lineage-specific active states in hESC, hNPC, or hME cells. Heatmaps are ordered by H3K4me1 signal at the enhancer within hESCs. **B** Heatmaps of ChIP-seq H3K27ac, H3K4me1, and H3K27me3 data within mESCs and of H3K27ac data within in vivo dissections of mECT, mEND, and mMES tissues for E7.5 embryos. Shown here are enhancer elements with lineage-specific active states in mESC cells, E7.5 mECT, or E7.5 mMES tissues. Heatmaps are ordered by H3K4me1 signal at the enhancer within mESCs. **A **and** B** Enhancer groups called as ePrimed (high H3K4me1, low H3K27ac), eNon-primed (low H3K4me1, low H3K27ac), or intermediate (intermediate H3K4me1) are displayed. **C** ATAC-seq profile for hNPC and hME called enhancer groups within in vitro primed hESCs. Running averages in 50-bp windows around the center of the enhancer (2 kb upstream and downstream) are shown alongside the averaged global signal (black dashed line). **D **and** E** DNA methylation levels over 500 bp core of active hESC enhancers (white) and hNPC and hME called enhancer subgroups: ePrimed (ep.) and eNon-primed (enp.) (**D**) by WGBS within in vitro primed hESC and (**E**) by scNMT-seq of in vivo human preimplantation embryos. **F** ATAC-seq profile for E7.5 mEct and E7.5 mMES called enhancer groups within in vitro mESCs. Running averages in 50-bp windows around the center of the enhancer (2 kb upstream and downstream) are shown alongside the averaged global signal (black dashed line). **G **and** H** DNA methylation levels over 500 bp core of active mESC enhancers (white) and mECT and mMES called enhancer subgroups: ePrimed (ep.) and eNon-primed (enp.) (**G**) by WGBS within in vitro naive mESC/mEpiLCs and (**H**) by scNMT-seq of in vivo mouse embryos at days E4.5 and E6.5. **D**,** E, G**, **H** Box plots show median levels and the first and third quartiles, whiskers show 1.5 × the interquartile range. Global methylation levels are displayed (red dashed line) along with results of ANOVA test between ePrimed and eNon-primed groups (***p < 0.001, *p < 0.05)

To identify epigenetic states of enhancer elements within the epiblast of human embryonic development, we analyzed publicly available epigenomic data collected from postgastrulation stage tissues differentiated from human epiblast-like embryonic stem cells (hESCs) [17]. Lineage-specific enhancer elements for hESC-derived mesendoderm (hME) and neural progenitor stem cells (hNPC) were called from a previous list of random-forest model-generated lineage-enriched enhancers [17], which were then subset by those which displayed lineage-specific H3K27ac (active enhancer) signals. This selection resulted in groups of predicted enhancer elements (1957 hNPC-specific and 4344 hME-specific enhancers) that appear epigenetically active only upon differentiation into their respective postgastrulation lineage (Fig. 1A). As a control group, we selected hESC enhancers with H3K27ac signal exclusively within the hESC lineage (i.e., absent in the postdifferentiation lineages), resulting in 3750 hESC-specific enhancer elements (Fig. 1A). We next looked at additional histone modification signatures within these lineage-specific enhancer groups and characterized their activity state in hESCs, categorizing them either as active (H3K27ac +/H3K4me1 +/H3K27me3-), poised (H3K27ac-/H3K4me1 +/H3K27me3 +), primed (H3K27ac-/H3K4me1 +/H3K27me3-), or inactive (H3K27ac-/H3K4me1-/H3K27me3-) (Additional file 1: Figs. S1-S3). We identified a substantial subset of hNPC-specific and hME-specific enhancers displaying a primed epigenetic signature within hESCs (27.4% of hNPC-specific enhancers/20.3% of hME enhancers) (Fig. 1A), which we hereafter refer to as epiblast primed (ePrimed) enhancers. For comparative analysis, we generated a similarly sized subset of enhancers from the Inactive group which had the lowest H3K4me1 signal within the hESCs (Fig. 1A), which we hereafter refer to as epiblast non-primed (eNon-primed). We note that while these eNon-primed enhancers are not epigenetically primed within the epiblast, they may still undergo priming ahead of their activation at a time point not captured within this dataset. We additionally identified a smaller subset of these lineage-specific enhancers which displayed the poised epigenetic signature within hESCs (Additional file 1: Fig. S4), which we shall refer to as epiblast poised (ePoised).

DNA methylation and chromatin accessibility analysis of ePrimed enhancer subsets within hESCs [18, 19] identified additional associated signatures, including increased chromatin accessibility (Fig. 1C), and decreased DNA methylation levels (Fig. 1D) when compared to eNon-primed enhancers from the same lineage group. Notably, single-cell multi-omic analysis (scNMT-seq) of in vivo preimplantation human embryos shows similar DNA hypomethylation states at ePrimed enhancer sites (Fig. 1E), suggesting that the epigenetic priming signature we observe within in vitro cultures recapitulates a regulatory program present within in vivo embryonic development. Interestingly, priming of lineage-specific enhancer elements was not observed to result in stronger activation of the enhancers upon differentiation, with little correlation between H3K4me1 signal in the hESC and the H3K27ac levels in the differentiated cell types (Additional file 1: Fig. S5A). However, hESC H3K4me1 levels did correlate with hESC H3K27ac levels, with ePrimed enhancers showing a weak enrichment of H3K27ac signal over eNon-primed enhancers (Additional file 1: Fig. S5B), albeit at significantly lower levels than those present at active enhancers. This could suggest that there is either mild or transient activation of ePrimed enhancers as part of establishment/maintenance of the primed enhancer state.

To identify lineage-specific enhancer histone modification dynamics in mouse embryonic enhancers and compare them to epigenetic states of lineage-specific enhancer elements in the mouse embryonic epiblast, we used existing lineage-specific promoter-distal H3K27ac data from E7.5 ectoderm (mECT), endoderm (mEND), and mesoderm (mMES) [16, 20], and then analyzed the epigenetic signatures of these sites within epiblast-like mouse embryonic stem cells (mESCs) in naive culture conditions [21] (Fig. 1B, Additional file 1: Fig. S3C-D, Fig. S6). Similar to our analysis of human enhancers, we identified a substantial subset of lineage-specific enhancers for all three germ layers that display a primed epigenetic signature (H3K27ac-/H3K4me1 +/H3K27me3-) in mESCs. Like the human ePrimed enhancers (Fig. 1C, D), the mouse ePrimed enhancers display increased chromatin accessibility and decreased DNA methylation levels, when compared to global levels and eNon-primed enhancers, both within in vitro culture and in vivo single-cell multi-omic datasets [16, 22, 23] (Fig. 1F–H, Additional file 1: Fig. S5). Additionally, we observed a similar weak enrichment of H3K27ac signal at H3K4me1 marked ePrimed enhancers over eNon-primed enhancers (Additional file 1: Fig. S5C, D). These results suggest that profiling of H3K4me1 is able to identify subsets of endoderm and mesoderm enhancers undergoing priming signatures which are otherwise obscured within prior class-wide chromatin accessibility and DNA methylation analyses [16]. Consistent with previous findings [16], these priming signatures are more pronounced within ectoderm and ectoderm-derived neural lineage enhancers, especially within later epiblast tissues (Fig. 1C, D, F, H), compared to those observed at endoderm/mesoderm lineage enhancers.

### Primed enhancers are associated with lineage-specific regulation of developmental gene networks

**Fig. 2:**
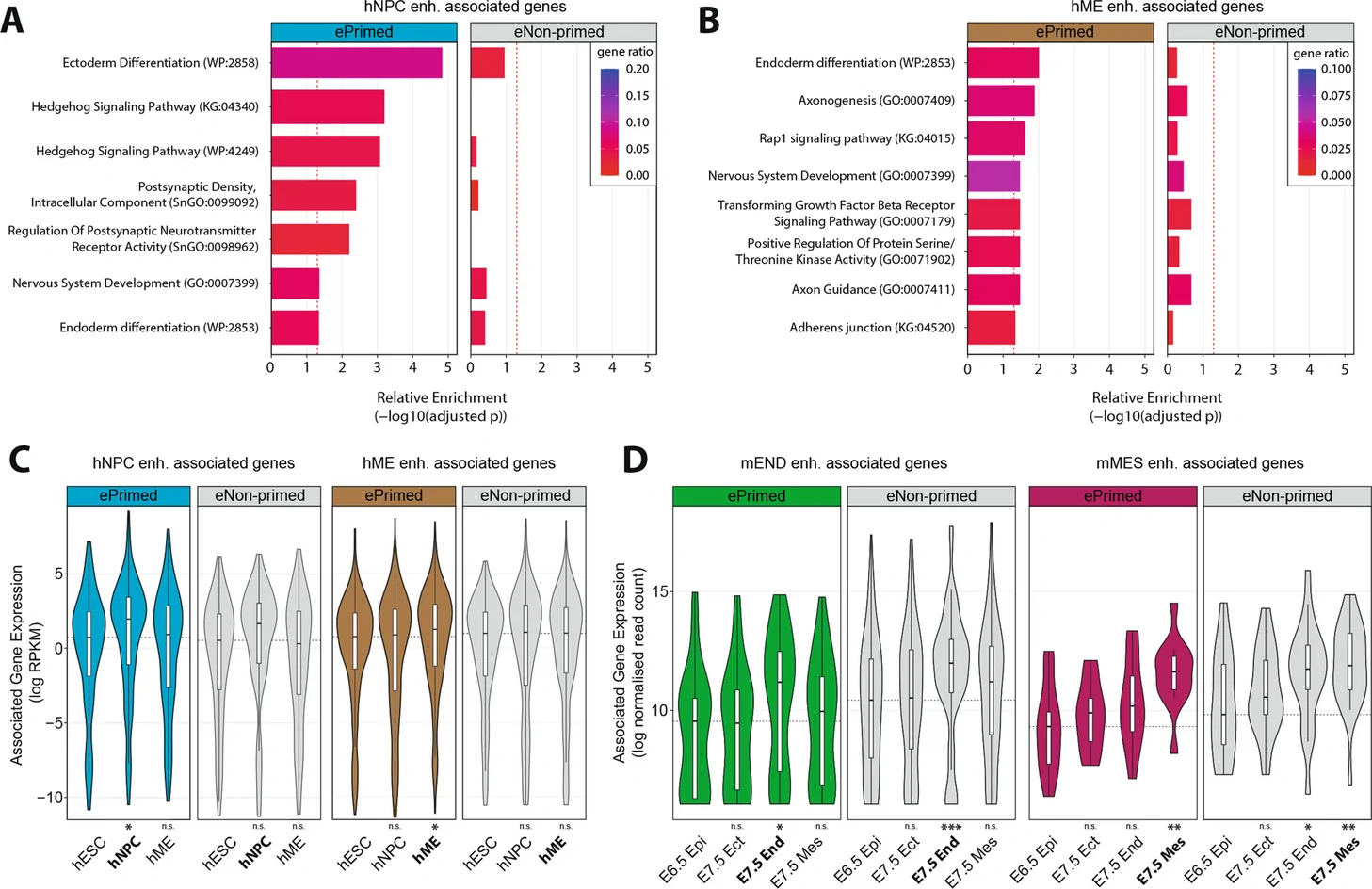
**A**,** B** Gene ontology enrichment analysis for ePrimed and eNon-primed enhancer-associated genes as defined by PCHiC/proximity hybrid model. A significance threshold of *p* < 0.05 is displayed (red dashed line). **A** Analysis of ePrimed and eNon-primed hNPC enhancer-associated genes and **B** ePrimed and eNon-primed hME enhancer-associated genes. **C**,** D** Overlaid box and violin plots show the expression of enhancer-associated genes. Box plots show median levels and the first and third quartile, whiskers show 1.5 × the interquartile range. Results from Welch’s two-sample *t* test against E6.5 epiblast/hESC are displayed (**p < 0.01, *p < 0.05). **C** Expression (log RPKM) within in vitro RNA-seq data collected from hESC, hNPC, hME cultures of genes associated exclusively with either ePrimed hNPC (blue) enhancers, eNon-primed hNPC (gray) enhancers, ePrimed hME (brown) enhancers, or eNon-primed hME (gray) enhancers. Median expression for each gene group within hESCs is also shown (gray dashed line). **D** Expression (log normalized counts) within in vivo scNMT-seq data collected from E6.5 epiblast and E7.5 ectoderm/endoderm/mesoderm tissues for genes associated exclusively with either ePrimed E7.5 mEND (green) enhancers, eNon-primed E7.5 mEND (gray) enhancers, ePrimed E7.5 mMES (purple) enhancers, or eNon-primed E7.5 mMES (gray) enhancers. Median expression for each gene group within E6.5 epiblast is also shown (gray dashed line)

To identify the impact of epigenetic priming of lineage enhancers on associated gene networks, we attempted to assign enhancers to the promoters they likely control. In the absence of chromatin conformation data from the postepiblast tissues we are investigating, we utilised a combination of genomic proximity models and Promoter Capture Hi-C data of mEpiLCs/hESCs [21, 24] to assign enhancers to promoters they likely interact with, as previous studies have shown that enhancers can interact with promoters prior to their activation [25, 26]. We then further subset these for genes found to be interacting with exclusively ePrimed or eNon-primed enhancer subgroups. To determine the functional role of gene networks associated with ePrimed enhancers, we performed a gene ontology enrichment analysis. This showed that ePrimed enhancers were enriched for associations with developmental gene networks (Fig. 2A, B). Genes exclusively associated with ePrimed hNPC enhancers are enriched for ectoderm differentiation and various neural development relevant processes such as Hedgehog signaling pathway and postsynaptic density (Fig. 2A). Conversely, eNon-primed hNPC enhancer exclusively associated genes were not found to be enriched for any specific biological processes. The ePrimed hME enhancer-associated genes are similarly enriched for tissue-relevant processes, with enrichments for endoderm differentiation genes along with signaling pathways and processes related to axon development, which are important for the mesoderm’s function in establishing the neural tube [27] (Fig. 2B). Again, hME eNon-primed enhancers were not enriched for any specific biological process. Similarly in mouse, ePrimed mECT/mEND enhancer-associated genes were enriched for morphogenic and developmental processes whereas eNon-primed mECT/mEND enhancer-associated genes were not enriched for any specific processes (Additional file 1: Fig. S6A, B).

Next, we analyzed the expression dynamics of these gene groups during human in vitro differentiation [17] and mouse in vivo gastrulation [16]. In the hESC differentiation model, genes exclusively associated with ePrimed hNPC enhancers were significantly upregulated (1.9-fold) upon differentiation into hNPCs (Fig. 2C). This upregulation appears to be lineage-specific to hNPC wherein the corresponding hNPC enhancer becomes marked by the active mark H3K27ac (Fig. 1A), with no observed upregulation upon differentiation into hMEs where the corresponding enhancer is not activated (Figs. 1A, 2C). Likewise, genes associated with primed hME enhancers are upregulated specifically in hME lineage differentiation (Fig. 2C). Comparable lineage-specific expression patterns were observed for genes associated with ePrimed enhancers during mouse gastrulation (Fig. 2D). Genes associated with ePrimed E7.5 mEND enhancers are upregulated specifically in E7.5 endoderm (Fig. 2D) coinciding with when the corresponding enhancer gains H3K27ac (Fig. 1B). This is specific to endoderm as the same patterns are not seen within E7.5 mesoderm or ectoderm tissues where the corresponding ePrimed E7.5 mEND enhancers do not gain these active marks (Figs. 1B, 2D). Similarly, genes associated with ePrimed mMES enhancers are significantly upregulated exclusively within the mesoderm at E7.5 (Fig. 2D). Conversely, the regulation of eNon-primed enhancer-associated gene groups is far less consistent, with many showing less lineage specificity in their expression. In the hESC differentiation model, genes exclusively associated with eNon-primed hNPC enhancers show an observable but statistically insignificant upregulation within hNPCs (Fig. 2C). In contrast, genes associated with eNon-primed hME enhancers show no expression changes upon differentiation from hESCs into hNPCs or hMEs (Fig. 2C). Some eNon-primed enhancer-associated genes do show lineage-specific expression, with eNon-primed E7.5 mEND enhancer-associated genes displaying significant upregulation exclusively within the mEND lineage at E7.5 (Fig. 2D). However, genes associated with eNon-primed E7.5 mMES enhancers are not lineage-specific in their expression and are significantly upregulated within both mesoderm and endoderm E7.5 tissues (Fig. 2D). Notably, we failed to detect significant upregulation of E7.5 mECT enhancer-associated genes, possibly as E6.5 epiblast already exists within a partial ectodermal state (Additional file 1: Fig. S7C). Interestingly, within the majority of somatic tissues, eNon-primed enhancer-associated genes were typically upregulated relative to ePrimed enhancer-associated genes, suggesting that they are enriched for non-lineage-specific postgastrulation processes (Additional file 1: Fig. S8). These observations suggest that the epigenetic priming signature of enhancers within the epiblast is associated with an increased lineage specificity in the regulation of associated gene networks, consistent with a central role for enhancer priming in the regulation of key developmental gene networks.

### A subset of early brain lineage-specific enhancers are epigenetically primed in the epiblast

**Fig. 3:**
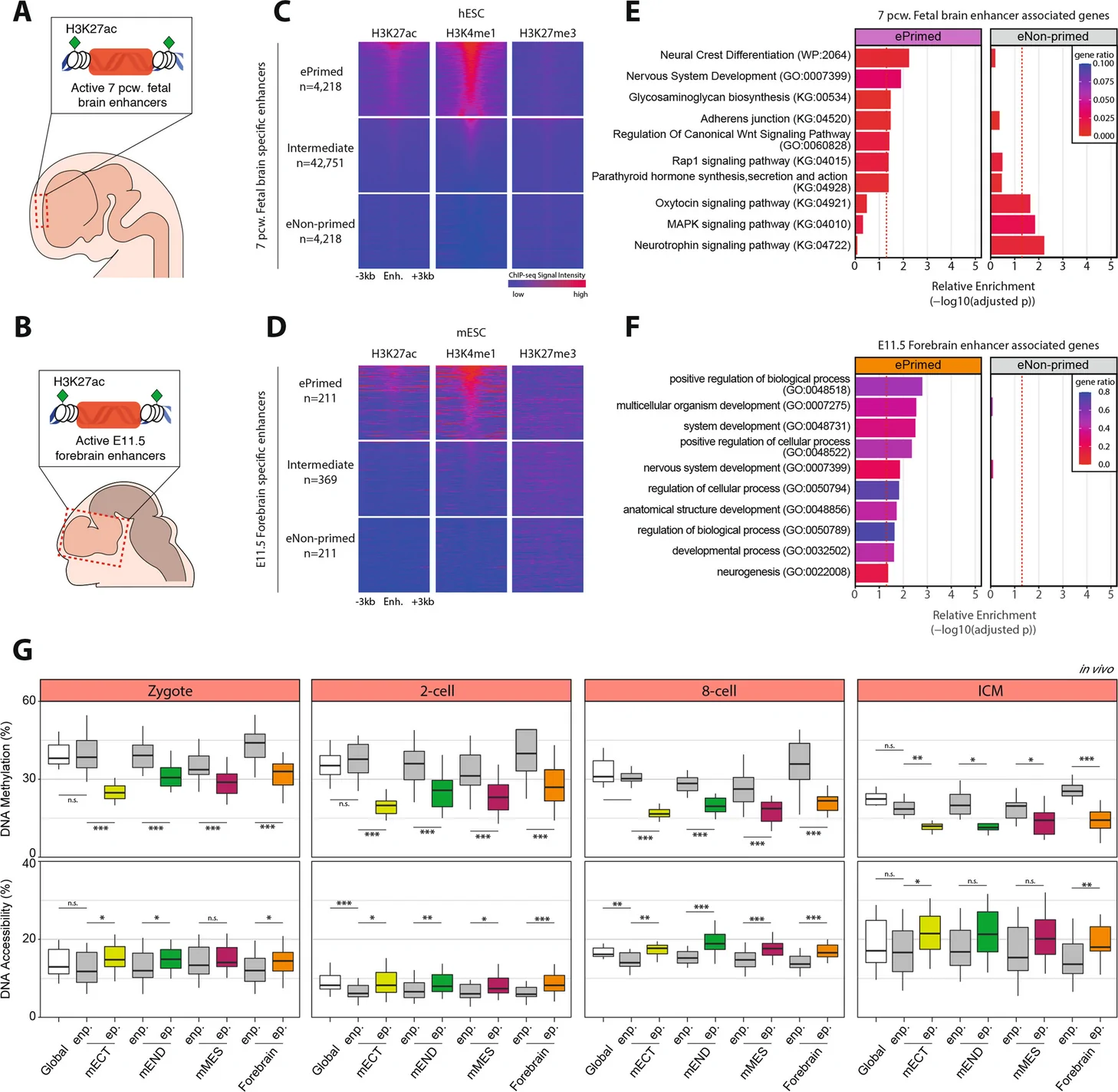
**A**, **B** Graphical representations of tissue dissections taken for H3K27ac ChIP-seq libraries for the identification of active enhancers within **A** human 7 postconception week late-embryonic brain and **B** mouse E11.5 forebrain. **C** Heatmaps of ChIP-seq H3K27ac, H3K4me1, and H3K27me3 data within hESC cells in culture at lineage-specific enhancers for 7pcw late-embryonic brain tissues that are called as ePrimed, eNon-primed, and intermediate states within the hESCs. **D** Heatmaps of ChIP-seq H3K27ac, H3K4me1, and H3K27me3 data within mESC cells in culture at lineage-specific enhancers for E11.5 forebrain tissues that are called as ePrimed, eNon-primed, and intermediate states within the mESCs. **E**,** F** Gene ontology enrichment analysis for **E** ePrimed and eNon-primed 7pcw late-embryonic brain enhancer-associated genes and **F** ePrimed and eNon-primed E11.5 forebrain enhancer-associated genes. Significance threshold of *p* < 0.05 is displayed (red dashed line). **G** COOL-seq analysis of DNA methylation and chromatin accessibility levels within preimplantation mouse embryos over 500 bp core of E7.5 mECT, E7.5 mEND, E7.5 mMES, and E11.5 forebrain called enhancer subgroups: ePrimed (ep.) and eNon-primed (enp.). Global levels for each cell are shown (white). Results for comparison of means between groups (ANOVA) are shown (***p < 0.001, **p < 0.01, *p < 0.05)

Curiously, we found that only a small proportion (< 1% in mouse and human) of ePrimed enhancers become active enhancers (i.e., gain H3K27ac) within tissues immediately derived from the epiblast. To understand if enhancer priming was also found at enhancers associated with later cell fate decisions, we next analyzed lineage-specific enhancers only activated at a significantly later embryonic developmental timepoint. Specifically, we focused on comparative neural tissues collected at E11.5 from mouse embryos [28] and 7 weeks postconception (7pcw) tissues from human embryos [29]. Peak calling of H3K27ac ChIP-seq data collected from E11.5 mouse forebrain identified 5583 distal peaks. These putative active enhancer elements were further filtered for lineage-specific enhancers by removal of sites with H3K27ac signal in earlier developmental tissues, which yielded 839 E11.5 forebrain-specific enhancers. As validation of our enhancer calling, we compared these enhancer sites to those within the VISTA Enhancer Browser [30]. Out of 183 mouse enhancers annotated with forebrain activity within the VISTA Enhancer Browser dataset, 155 overlap our putative E11.5 forebrain enhancers, 8 of which overlap enhancers that are active exclusively within the forebrain (Additional file 1: Fig S9A, B). Similarly, we called 77,235 distal H3K27ac peaks within human 7pcw late-embryonic brain tissues, 51,187 of which were lineage-specific, with no evidence of H3K27ac signal within the aforementioned earlier tissues analyzed. Strikingly, there are 4218 7pcw late-embryonic brain ePrimed enhancers (Fig. 3A, C) and 211 E11.5 forebrain ePrimed enhancers (Fig. 3B, D) that are found to be in an epigenetically primed state (i.e., H3K4me1 positive) already within the epiblast. These later-development ePrimed enhancers show similar H3K4me1 signals in the absence of poised enhancer-associated H3K27me3 or active enhancer-associated levels of H3K27ac. Similar to what we observed at the other ePrimed enhancers described above, there is a weak H3K27ac signal at a subset of 7pcw late-embryonic brain and E11.5 forebrain ePrimed enhancers, although the signal is much lower than at active hESC/mESC enhancers (Fig. 3C, D). Furthermore, E11.5 forebrain and 7pcw late-embryonic brain ePrimed enhancers were also associated with decreased DNA methylation and increased chromatin accessibility (Additional file 1: Fig. 10A-D). Gene ontology analysis of E11.5 forebrain and 7pcw late-embryonic brain ePrimed enhancer-associated genes showed significant enrichment for key nervous system developmental processes and neurogenesis (Fig. 3E, F). In contrast, eNon-primed enhancer-associated genes were generally not enriched for neurodevelopmental processes (Fig. 3E, F). Protein–protein association network analysis by STRING [31] shows that 7pcw brain ePrimed enhancer-associated transcription factors are significantly interconnected with enrichment for TFs that coordinate head development, brain development, neuron fate commitment, and regulation of neuron differentiation (Additional file 1: Fig. S11).

Importantly, epigenetic enhancer priming within the epiblast for later developmental tissues is not exclusive to neural-lineage regulatory elements. We identified similar subsets of late-embryonic (6–8 postconception weeks) human liver and kidney enhancers and E11.5 mouse liver and heart enhancers, which were epigenetically primed within the epiblast (Additional file 1: Figs. S12&13). Similarly, these ePrimed enhancer groups were found to be associated with genes and TF networks enriched for developmental processes (Additional file 1: Fig. S11C, Fig. S12C&F). Some developmental gene networks appear to be regulated by ePrimed enhancers in both human and mouse (Additional file 1: Fig. S14A-D), such as Wnt signaling genes associated with ePrimed brain enhancers (Additional file 1: Fig. S14B). However, there is little conservation of the ePrimed enhancers and the genes they potentially regulate (Additional file 1: Fig. S14A,C,E–F). Overall, our findings suggest that epigenetic priming not only occurs at enhancers that will be activated in immediately following developmental stages but also at enhancers that become active at much later stages.

### Epigenetic priming of enhancers originates in the zygote

To study when epigenetic priming of ePrimed enhancers is first established, we analyzed single-cell multi-omics sequencing data from the initial stages of mouse embryogenesis ranging from the zygote to the early blastocyst captured by COOL-seq, a sequencing technology that can simultaneously analyze chromatin accessibility, DNA methylation, and DNA copy number variation for individual mammalian cells [32]. Surprisingly, we observed that E7.5 germ-layer and E11.5 forebrain ePrimed enhancers were hypomethylated as early as the fertilized zygote and remained hypomethylated in the inner cell mass of the blastocyst (Fig. 3G). Similarly, we found that chromatin at ePrimed enhancers is more accessible than at eNon-primed enhancers in the zygote, which further increases upon the establishment of the ICM (Fig. 3G).

As reduced levels of DNA methylation marks were consistently observed at primed enhancer sites, we next looked at the dependence of this pattern on the enzymes that are involved in the establishment and removal of DNA methylation marks. DNA methyltransferases (DNMTs) are responsible for de novo methylation (DNMT3a/DNMT3b) [33], while 10–11 translocation proteins (TETs) facilitate the removal of DNA methylation marks by oxidation [34–36]. We analyzed DNA methylation levels of hESC cell lines with combinations of genetic knockouts for DNMTs and TETs [37]. In DNMT3A/DNMT3B double knockouts, primed enhancers show a significant decrease in DNA methylation (Additional file 1: Fig. S10E). In TET1/2/3 triple knockout cells, there is a loss of the hypomethylation signature at primed enhancers, which is then recovered when DNMT3A/DNMT3B are knocked out simultaneously (Additional file 1: Fig. S10E). These results suggest that the DNA hypomethylation at ePrimed enhancers in hESCs is maintained by TET/DNMT antagonistic activity, similar to the dynamics and antagonistic activity that occurs at active enhancers but not at eNon-primed enhancer sites (Additional file 1: Fig. S10E).

Neither early nor later developmental ePrimed enhancers appear to be silenced by DNA methylation or loss of accessibility immediately upon a cell fate transition into an alternative lineage (Additional file 1: Fig. S15A). Notably, most ePrimed and ePoised enhancers also initially maintain their H3K4me1/H3K27me3 modifications upon differentiation towards an alternative lineage (Additional file 1: Fig. S15B). Argelaguet et al. [16] showed that primed ectoderm lineage enhancers exhibit DNA hypomethylation in nonectodermal germ layers, suggesting an epigenetic memory of the priming event. We observed a similar epigenetic memory of the priming event within our ePrimed enhancer groups, with DNA hypomethylation persisting in germ layer tissues in which the enhancer had not been activated. For example, ePrimed mEND enhancers remained hypomethylated when cells differentiated into ectoderm and mesoderm (Additional file 1: Fig. S10F). Notably, we observed that the hypomethylation signature at ePrimed enhancers persisted into adult somatic tissues, similarly regardless of the germ-layer origin of the tissue (Additional file 1: Fig. S16).

### Defining sequence determinants of ePrimed enhancers using naturally occurring regulatory human genetic variation

**Fig. 4:**
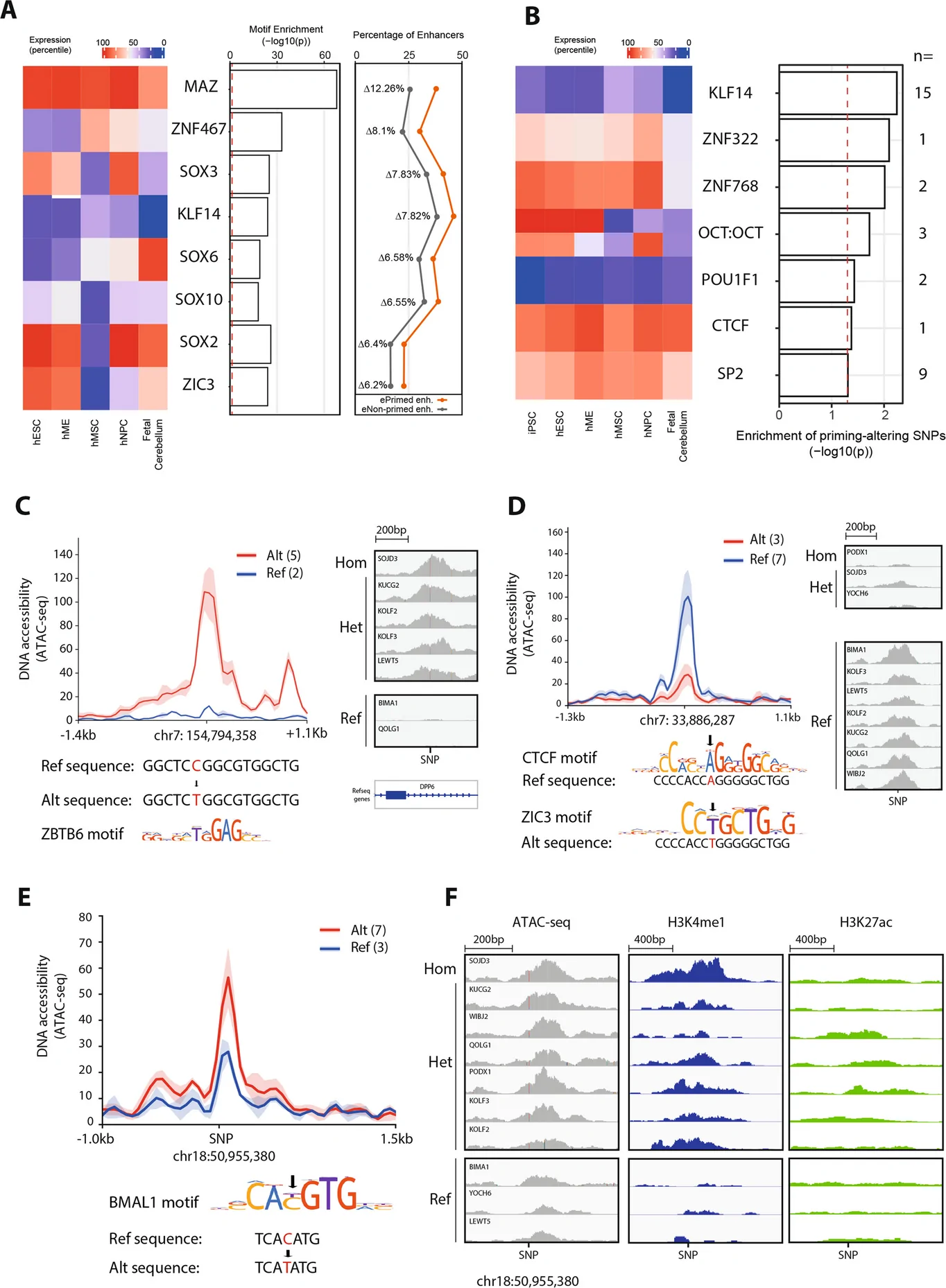
**A** Motif enrichment analysis of human ePrimed versus eNon-primed 7pcw late-embryonic brain enhancers. Heatmap of relative expression (percentile rank) of the significantly enriched motif elements within hESC, hME, hMSC, hNPC cultures, and in vivo late-embryonic cerebellum tissue (collected 72–129 postconception days). Barplot shows *p* values for the motif enrichment test with a significance threshold of *p* < 0.05 displayed (red dashed line). Line plot shows the percentage of enhancers which contain an instance of each motif for ePrimed (orange) and eNon-primed (gray) 7pcw late-embryonic brain enhancer subgroups. Points are annotated with the percentage differential between ePrimed and eNon-primed subgroups. **B** Motifs associated with priming-altering SNPs within HipSci iPSC lines. Heatmap of relative expression (percentile rank) of the significantly enriched motif elements within hESC, hME, hMSC, hNPC cultures, and in vivo late-embryonic cerebellum tissue. Expression of OCT4 and OCT6 is shown for OCT:OCT. Barplot shows *p* values of the association test between disruption of priming and presence of SNP within the motif sequence (Fisher’s exact test) with a significance threshold of *p* < 0.05 displayed (red dashed line). **C–E** Profiles of chromatin accessibility and genome browser visualization of priming-altering associated SNP examples within ePrimed 7pcw late-embryonic brain enhancers. HipSci donors are grouped based on the sequence variant. Running averages in 50 bp windows around the SNP are shown. Solid lines show the mean across donor lines and shaded areas represent the standard deviation. Genome browser visualization of ATAC-seq (gray) signal within the donor lines, grouped by allelic presence of SNP. **C** Example of introduction of ZBTB6 motif by C → T SNP at chr7:154,794,358 (hg38). Donor lines containing a second confounding SNP were excluded. **D** Example of altering CTCF motif to ZIC3 motif by A → T SNP at chr7:33,886,287 (hg38). **E** Example of altering a BMAL1 motif by C → T SNP at chr18:50,955,380. **F** Genome browser visualization of ATAC-seq (gray), H3K4me1 (blue), and H3K27ac (green) signal within the donor lines, grouped by allelic presence of SNP chr18:50,955,380 (hg38)

Having identified groups of ePrimed and eNon-primed enhancers, we next asked if the DNA sequence composition of primed enhancers could contribute to their epigenetic priming. Since previous studies have shown associations with H3K4me1 marks and the binding of pioneer factors [38–40], we performed motif enrichment analysis. This revealed enrichment of various TF motifs within ePrimed 7pcw late-embryonic brain enhancer regions when compared to eNon-primed counterparts. The enriched motifs include expected neural development-associated TFs, such as MAZ [41] and SOX6 [42] (Fig. 4A). The ePrimed enhancer enriched motif sequences also include motifs for TFs which are more highly expressed at epiblast-like stages, such as SOX3, SOX2, and ZIC3, which could be involved in the establishment or maintenance of the primed state (Fig. 4A). However these motifs are not exclusive to ePrimed enhancers as they are also found prevalent within their respective non-primed counterparts (Fig. 4A).

We next used human-induced pluripotent stem cell lines from different donors to functionally interrogate TF motif sequences and their respective importance for the observed epigenetic priming signatures. The human-induced pluripotency stem cell initiative (HipSci) has derived induced pluripotent stem cells (iPSC) from skin fibroblasts taken from a number of patients and healthy donors containing a collection of germline inherited and somatically acquired polymorphisms [43, 44]. This naturally occurring genetic variation can be used to query the relationship between primary sequence and enhancer priming. We performed ATAC-seq [45] and CUT&Tag [46] for H3K4me1 and H3K27ac in 10 HipSci iPSC lines, representing 9 unique healthy donors. Many of our primed 7pcw brain enhancers coincided with chromatin accessibility peaks and H3K4me1 signal in the absence of H3K27ac, suggesting that these iPSC lines recapitulate the priming state observed in the epiblast at many of these enhancers (Additional file 1: Fig. S17A, B). We next identified single nucleotide polymorphisms (SNPs) which overlapped TF motifs in these primed enhancers and identified SNPs that disrupted their priming by identifying either a gain or loss of ATAC-seq peak when an SNP was present. For 145 different TF motifs overlapping a variant in an enhancer with altered accessibility, 7 TF motifs were significantly enriched (KLF14, ZNF322, ZNF768, OCT:OCT, POU1F1, CTCF, SP2) (Fig. 4B).

We observed three different scenarios of how particular SNPs can alter TF motif sequences. First, we found instances where SNPs would introduce/ablate a motif entirely, such as the introduction of a ZBTB6 motif which coincided with a marked increase in priming of the associated enhancer (Fig. 4C). Second, we observed instances where an SNP would alter a key residue within a motif and result in the introduction of an alternative TF motif, such as the replacement of a CTCF motif for a ZIC3 motif which resulted in a loss of associated priming state (Fig. 4D). Lastly, we observed instances where a SNP appears to alter the preferred base within the motif towards a less preferred base, potentially weakening the binding affinity. One example was the change of a preferred C residue to a weaker affinity T base within the motif for the BMAL1 pioneer-like TF factor; this potential weaker affinity motif was associated with an increase of priming signature at the associated enhancer, with both increased DNA accessibility and H3K4me1 signal (Fig. 4E, F). This could be caused by preferences in degenerate motifs for pioneer factors when targeting nucleosome-bound DNA [47]. Our results demonstrate that while motif enrichment analysis is unable to identify individual sequences that are predictive for enhancer priming, naturally occurring genetic variation can be integrated with epigenetic profiling to reveal potentially important enhancer motifs.

## Discussion

This study has identified a program of enhancer priming in human embryo development similar to that previously observed associated with mouse gastrulation [16] indicative of an evolutionarily conserved mammalian program for epigenetic priming of lineage-specific regulatory elements. Priming signatures are more pronounced in ectoderm lineage enhancers, especially in late epiblast tissues; however, our findings suggest enhancer priming is used to also establish the capacity for differentiation into the mesoderm and endoderm fates. Similar to enrichments of poised enhancer-associated gene networks [8], this conserved program of enhancer priming appears to be linked with the lineage-specific regulation of key developmental gene networks, pointing to an important role in healthy embryonic development.

What remains unclear is the functional impact of epigenetic priming of these enhancers, whether their priming is required for accurate and rapid activation of associated genes to control developmental gene networks as well as the functional importance of the priming associated epigenetic marks themselves. While the H3K4me1 mark identifies these preactive state enhancers, recent studies have begun to question if the mark itself or associated histone modifiers are responsible for driving enhancer activation and subsequent transcriptional responses. Many enhancer elements, including key regulators of early differentiation, still gain H3K27ac upon knockout of H3K4me1 histone modifiers MLL3 and MLL4 [48]; however, conversely, many enhancers lose the ability to become activated upon MLL4 KO [49]. These conflicting results suggest a potential context dependency for the importance of H3K4me1 and its writers. However, these KO studies are unable to distinguish between the importance of the H3K4me1 mark and the potential enzymatic activity-independent functions of its writers who themselves may recruit the transcriptional machinery directly.

Questions also remain regarding the temporal order and interplay between the various epigenetic marks associated with the primed enhancer state. Interestingly, our observations reveal that primed enhancers are hypomethylated and show nucleosome displacement as early as the zygote. It is therefore possible that these marks precede and may be required to recruit the observed H3K4me1 signature. DNA hypomethylation is indeed observed developmentally early, in the zygote, and may facilitate the recruitment of TFs that subsequently recruit H3K4 methyltransferases [50–53]. Conversely, it has been hypothesized that the H3K4me1 reader, TIP60, can promote the integration of H2A.Z-containing nucleosomes to create more dynamic and open chromatin structures [54, 55]. These hypotheses could also point towards a positive feedback loop between these epigenetic layers and may explain why the priming signature is stable enough to persist for long periods of development and many cell divisions as observed in our models and previous observations of stable H3K4me1 marked regions during zebrafish development [56]. Capture and analysis of H3K4me1 datasets of earlier developmental time points would be required to dissect the relative timings of these epigenetic marks, while targeted epigenetic manipulation of ePrimed and eNon-primed enhancers in differentiation models in combination with inducible degron in vivo models could help dissect the respective roles and interplay between these epigenetic marks.

Another important question is how priming of the particular enhancer regions is initiated during development. Our study identified a number of enriched sequence motifs but no universal priming-associated enhancer sequence signature. A limitation of our study is that the analysis has not considered the relative strength of the motifs present or whether combinations of motifs may predict sites which undergo epigenetic priming. Furthermore, the identified enriched motifs will require further experimental interrogation to validate their functional role. Additionally, this analysis is limited by the natural variation within the donor lines used; the power of this analysis could be further increased by the inclusion of additional lines and therefore increased SNP instances within the enhancer regions of interest. The inability to identify singular priming-associated motif sequences could suggest that enhancer priming is a complex process, defined by various sequence signatures in concert interplaying with additional factors such as the enhancer’s location within the genome. In this context, it is worth noting that enhancers have been shown to tolerate substantial sequence changes [57].

Interestingly, we did not observe rapid decommissioning of primed enhancers, with hypomethylation “scars” persisting at primed enhancers into adult somatic tissues. Previous studies have shown that developmental enhancers with hypomethylation scars retain the capacity to be reactivated upon PRC2 disruption [58] and suggested that the reactivation of developmental enhancers facilitates the regeneration of tissues in response to damage [59]. It is possible that hypomethylation scars at primed enhancers provide an epigenetic memory of developmental cell states, facilitating the return to these states upon perturbations required for the repair or rejuvenation of tissues. This would provide an interesting model to interrogate the importance of enhancer priming and maintenance of their epigenetic memory for the capacity of somatic cells to rejuvenate in perturbations such as Yamanaka factor-induced cellular rejuvenation [60].

## Conclusions

In this study, we perform multi-omic profiling of human and mouse embryonic development to determine the dynamics of enhancer epigenetic priming and to investigate its role in regulating cell fate decisions. We further leverage naturally occurring genetic variation in human pluripotent stem cell lines to identify sequence determinants and potential trans-acting factors that establish the primed state at key human developmental enhancers. Our work provides evidence of a conserved program of epigenetic priming for lineage-specific enhancers that exists in early human embryo development and demonstrates that epigenetic priming is present at enhancer elements for all three germ layers in both human and mouse epiblast tissues. The results of this study suggest that the observed epigenetic priming of enhancers is associated with the lineage-specific regulation of key developmental gene networks. Our findings that epigenetic priming within the epiblast also occurs at enhancer elements specific for later developmental stages (E11.5 in mouse and 7 weeks postconception in human) also suggest that enhancer priming occurs far earlier than previously anticipated and that its functional consequences manifest through remarkably long developmental periods along cell specification trajectories.

## Methods

### Human iPSC cell culture

Human-induced pluripotent stem cell (hiPSC) lines (HPSI1113i-bima_1, HPSI0114i-kolf_2, HPSI0114i-kolf_3, HPSI0214i-kucg_2, HPSI0514i-letw_5, HPSI1113i-podx_1, HPSI1113i-qolg_1, HPSI0314i-sojd_3, HPSI0214i-wibj_2, and HPSI0215i-yoch_6) were purchased from the Human Induced Pluripotent Stem Cells Initiative (HipSci; https://www.hipsci.org/), deposited by the Wellcome Trust Sanger Institute (https://www.sanger.ac.uk/), and banked at European Collection of Authenticated Cell Cultures (ECACC). ECACC’s quality control testing methods are accredited in accordance with the recognized International Standard to ISO/IEC 17025:2005. All hiPSC lines were purchased from Culture Collections of the UK Health Security Agency (https://www.culturecollections.org.uk/) and tested regularly for mycoplasma contamination.

All cell lines were cultured in TeSR-E8 media complete with supplement (Stemcell Technologies; 05990) on plates coated with vitronectin (Thermo Fisher Scientific; A14700) at 37 °C under 5% CO2 and normal O2 levels. Complete media was refreshed every 24 h. For the first day after revival, 10 µM rho-associated protein kinase (ROCK) inhibitor (Cell Guidance Systems; Y-27632) was added to the media. Cells were passaged at ratios ranging from 1/6 to 1/8 using 0.5 mM EDTA (Life Technologies; AM9260G) upon reaching approximately 70% confluency. Regular tests ensured that all HiPSC lines were negative for mycoplasma contamination.

### ATAC-seq

ATAC-seq libraries were generated as described in Corces et al.’s study [61], with minor modifications. After washing once with 1 × DPBS (Life Technologies; 14,190,144), hiPSCs were harvested with accutase (StemCell Technologies; 07922) by incubation for 5 min at 37 °C. After accutase neutralization with TeSR-E8 medium cells were centrifuged at 300 × *g* for 3 min at RT. After resuspension in 1 × DPBS, 50,000 cells underwent centrifugation at 500 × *g* for 5 min at 4 °C in a swing arm rotor centrifuge, then lysis in 50 µl of cold ATAC resuspension buffer (10 mM Tris–HCl pH 7.5, 10 mM NaCl, 3 mM MgCl2) containing 0.1% IGEPAL-630, 0.1% Tween-20, and 0.01% digitonin for 3 min on ice. The lysate was then topped up with 1 ml cold ATAC resuspension buffer containing 0.1% Tween-20 and centrifuged at 500 × *g* for 10 min at 4 °C in a swing arm rotor centrifuge. The pellet was then resuspended in 50 µl of transposition mixture (1 × Illumina Tagment DNA Buffer and 100 nM Illumina TDE1 Tagment DNA Enzyme transposase (Illumina; 20,034,197), 0.33 × DPBS, 0.1% Tween-20, 0.01% digitonin) and incubated at 37 °C for 30 min with 1000 RPM mixing.

After undergoing purification with the Zymo DNA Clean and Concentrator-5 kit (Zymo Research; D4003), samples were eluted in 21 µl of elution buffer and combined with 2.5 µl of 25 µM i5 primer, 2.5 µl of 25 µM i7 primer, and 25 µl 2 × NEBNext High-Fidelity 2 × PCR Master Mix (NEB; M0541S). A PCR was then performed with the following conditions: 72 °C for 5 min, 98 °C for 30 s, 8 cycles of 98 °C for 10 s, 63 °C for 30 s, and 72 °C for 1 min. The samples then underwent a second Zymo DNA Clean and Concentrator-5 purification and were eluted in 30 µl of elution buffer prior to a 1.2 × AMPure XP bead (Beckman Coulter; A63881) cleanup. Following QC on a Bioanalyzer, libraries were multiplexed and sequenced (paired-end 50 bp) using a HiSeq 2000 instrument (Illumina).

### CUT&Tag

CUT&Tag libraries were generated according to the EpiCypher protocol [46] with minor modifications. As described for ATAC-seq, hiPSCs were harvested with accutase (StemCell Technologies; 07922). After resuspension in 1 × DPBS (Life Technologies; 14,190,144), 2 × 10^5^ cells per CUT&Tag reaction underwent centrifugation at 300 × *g* for 3 min at RT. Cells were then lysed in 100 μl of cold nuclear extraction (NE) buffer (20 mM HEPES–KOH, pH 7.9, 10 mM KCl, 0.1% Triton X-100, 20% Glycerol, 0.5 mM Spermidine (Sigma-Aldrich; 05292), 1 × cOmplete™, Mini, EDTA-free Protease Inhibitor (Roche; 11,836,170,001)) per CUT&Tag reaction for 10 min on ice. The nuclei were then centrifuged for 3 min at 600 × *g* at RT before resuspension in cold NE Buffer to achieve a final concentration of 1.2 × 10^6^ nuclei/ml.

For each CUT&Tag reaction, 11 μl of concanavalin A (ConA) beads (EpiCypher; 21–1401) was washed twice on a magnetic stand with 100 μl of bead activation (BA) buffer (20 mM HEPES, pH 7.9, 10 mM KCl, 1 mM CaCl_2_, 1 mM MnCl_2_). The beads were then resuspended in 11 μl of BA buffer, and 10 μl beads were aliquoted into a 0.2-ml tube. Each tube of activated ConA beads was then incubated with 100 μl of nuclei for 10 min at RT. The supernatant was then removed with a magnet and the beads were resuspended in 50 μl of cold antibody150 buffer (20 mM HEPES, pH 7.5, 150 mM NaCl, 0.5 mM Spermidine, 1 × cOmplete™, Mini, EDTA-free Protease Inhibitor, 0.01% digitonin (Sigma-Aldrich, D141-100MG), 2 mM EDTA) containing a 1:50 dilution of rabbit primary antibody (anti-H3K4me1 (Active Motif; 39,298) or anti-H3K27ac (Abcam; ab4729)) and incubated overnight at 4 °C on a rocker.

The following day, the supernatant was discarded with a magnet and the beads were incubated in 50 μl of Digitonin150 buffer (20 mM HEPES, pH 7.5, 150 mM NaCl, 0.5 mM Spermidine, 1 × cOmplete™, Mini, EDTA-free protease inhibitor, 0.01% digitonin) containing 0.5 μg of anti-rabbit secondary antibody (Epicypher; 13–0047) for 1 h at RT. The beads were then washed twice on a magnet with Digitonin150 buffer, then incubated in 50 μl of Digitonin300 buffer (20 mM HEPES, pH 7.5, 300 mM NaCl, 0.5 mM Spermidine, 1 × cOmplete™, Mini, EDTA-free protease inhibitor, 0.01% digitonin), and 1.25 μl of CUTANA pAG-TN5 (EpiCypher; 15–1017) for 1 h at RT. The beads were then washed twice with a magnet by resuspension in Digitonin300 buffer, then incubated in 50 μl of chilled tagmentation buffer (Digitonin 300 Buffer, 10 mM MgCl_2_) for 1 h at 37 °C. The supernatant was then discarded with a magnet, and the beads were washed once with 50 μl RT TAPS buffer (10 mM TAPS, pH 8.5, 0.2 mM EDTA). The supernatant was again removed with the magnet and the beads were resuspended in 5 μl of RT SDS release buffer (10 mM TAPS, pH 8.5, 0.1% SDS) and vortexed on maximum speed for 10 s followed by a brief centrifugation. The beads were then incubated for 1 h at 58 °C before the addition of 15 μl of RT SDS Quench Buffer (0.67% Triton-X 100 in molecular grade H_2_O), vortexing at maximum speed for 10 s, and a brief centrifugation.

For library amplification, samples were combined with 2 μl of 5 µM i5 primer, 2 μl of 5 µM i7 primer, and 25 μl of CUTANA high fidelity PCR mix (EpiCypher; 15–1018). The following PCR program was then used for all libraries: 58 °C for 5 min, 72 °C for 5 min, and 98 °C for 45 s. This was followed by 11 cycles for anti-H3K27ac and 13 cycles for anti-H3K4me1 of 98 °C for 15 s and 60 °C for 10 s, before a final extension at 72 °C for 1 min. Libraries then underwent two 1 × AMPure XP bead (Beckman Coulter; A63881) cleanups. Following QC on a Bioanalyzer, libraries were multiplexed and sequenced (paired-end 150 bp) using a NovaSeq 6000 instrument (Illumina).

### Human preimplantation embryo scNMT-seq

The use of human embryos for this work has been approved by the Multi-Centre Research Ethics Committee and licensed by the Human Fertilization and Embryology Authority of the UK, under Research License R0178. Embryos from embryonic days 5–6 were collected in Corujo-Simon et al. [62] in which scRNA-seq was performed using the RNA half of the scNMT-seq method [63]. In this study, we used the gDNA from the same cells to generate DNA methylation and chromatin accessibility data using the published scNMT-seq protocol [64]. Briefly, genomic DNA was purified using AMPure XP beads then bisulfite converted with the Zymo EZ-96 DNA Methylation-Direct MagPrep kit. Sequencing libraries were then prepared from bisulfite-converted fragments via random primed synthesis using oligos containing Illumina primer sequences, followed by indexing PCR. Pooled libraries were sequenced using an Illumina HiSeq 2000 instrument using 125 bp paired-end reads (day 5 and day 6 embryos). Fastq files were aligned to the GRCh38 build of the human genome, and CpG methylation and GpC accessibility files were generated as previously described [16].

### Defining lineage-specific enhancer states within the epiblast

Murine ChIP-seq data for mESC in vitro cultures were obtained from Parry et al. 2023 [21] (GSE223569) [65], in vivo E7.5 tissues from GSE125318 [66], and in vivo late-embryonic tissues from the ENCODE project [28]. We downloaded the call sets from the ENCODE portal [67] (https://www.encodeproject.org/) with the following identifiers: ENCFF001ZRF, ENCFF001ZRD, ENCFF001ZRB, ENCFF001ZRL, ENCFF001ZRG, and ENCFF001ZRE. Reads were trimmed using Trim Galore v0.6.1 (using Cutadapt v1.18) [68] and mapped to *Mus musculus* GRCm38 using Bowtie2 v2.4.1 [69]. Human ChIP-seq data for primed hESC in vitro cultures were obtained from SRP000941 [70], for in vivo 7pcw late-embryonic brain tissue from GSE63648 [71], and for in vivo late-embryonic liver and kidney tissue from EGAS00001003163 [72, 73]. Reads were trimmed using Trim Galore v0.6.1 (using Cutadapt v1.18) and mapped to *Homo sapiens* GRCh38 using Bowtie2 v2.4.1 [69]. Quantification of these marks was performed using SeqMonk v1.48.2 [74]. H3K27ac peaks were called using an integrated MACS pipeline (*p* value cutoff 1.0E-5).

For early development human enhancers (hME, hMSC, hNPC), lineage-specific enhancers were obtained by excluding previously annotated enhancers from Xie et al. [17] and intersected with called hESC, hME, hMSC, and hNPC H3K27ac peaks using BEDtools intersect [75], subsetting for enhancer instances that failed to overlap H3K27ac peaks in tissues outside of their specific lineage. 7pcw late-embryonic brain, liver, and kidney lineage-specific enhancers were defined as H3K27ac peaks within the tissue that do not overlap with an annotated TSS (± 200 bp) or an ulterior-lineage H3K27ac peak (hESC, hMSC, hME, hNPC). For mouse enhancers, E7.5 mECT/mEND/mMES lineage-specific enhancers were defined as respective H3K27ac peaks that do not overlap with an annotated TSS (± 200 bp) or an ulterior-lineage H3K27ac peak. E11.5 lineage-specific enhancers were similarly defined as respective H3K27ac peaks that do not overlap with an annotated TSS (± 200 bp) or an ulterior E11.5, E7.5, or mESC lineage H3K27ac peak. logRPKM counts for ChIP-seq reads were quantified over 1500 bp probe centered on the enhancer region. Enhancer states within human epiblast were sequentially defined using these counts: active enhancers as regions with H3K27ac > 2.97, poised as remaining regions with H3K27me3 > 2, primed as remaining regions with H3K4me1 > 1.2, and epiblast-inactive as all remaining regions. Enhancer states within mouse epiblast were similarly defined as active enhancers with H3K27ac > 1.2, poised with H3K27me3 > 1.25, primed with H3K4me1 ≥ 0.4, and epiblast-inactive for remaining regions. For visualization of ChIP-seq, signal heatmaps were generated using Homer tools v4.11 [38] and Samtools v1.11 [76].

### Chromatin accessibility and DNA methylation at enhancers

ATAC-seq data for mESC in vitro cultures were obtained from GSE81679 [77] and whole-genome bisulfite (WGBS) data from PRJDB3812 [78]. Reads were trimmed using Trim Galore v0.6.6 (using Cutadapt v2.3) and mapped to *Mus musculus* GRCm38 using Bismark v0.23.0 [79]. ATAC-seq data for hESC cultures were obtained from GSE101074 [80] and WGBS data from GSE75868 [81]. WGBS data for hESC cultures with TET and DNMT combinatorial knockouts were obtained from GSE126958 [82]. WGBS data for human somatic tissues were obtained from the NIH Roadmap Epigenetics Consortium [83] and GSE16256 [84]. Reads were trimmed using Trim Galore v0.6.6 (using Cutadapt v2.3) and mapped to *Homo sapiens* GRCh38 using Bismark v0.23.0. For the visualization of ATAC-seq signals, Homertools v4.11 annotatepeaks function was used over a ± 3 kb region centered on the enhancer region. Quantitation of DNA methylation in vitro datasets was performed using Seqmonk Bisulfite methylation quantitation function over a 500 bp probe centered on the enhancer. Global levels of methylation are calculated as the mean of 10 kb running windows. Analysis of scNMT and COOL-seq data was performed using methods and scripts outlined in Argelaguet et al. [16].

### Analysis of enhancer-associated gene networks

Significant Promoter Capture Hi-C interactions within mESCs were obtained from GSE223578 [85] and within hESCs from GSE86821 [86]. Enhancers overlapping promoter-interacting fragments were identified by BEDtools intersect; proximal promoters within 20 kb of enhancers were also considered potentially interacting. Promoters were filtered for those interacting with enhancers from a singular lineage and enhancer state. Gene ontology enrichments were performed using the enrichr R package v3.2 (github.com/wjawaid/enrichR). Gene expression data for hESC, hME, hMSC, hNPC, and in vitro cultures were obtained from SRP000941 [70], for human late-embryonic tissues from GSE156793 [87, 88], and for human somatic tissues from GSE144530 [89, 90]. Expression of protein-coding genes was converted to percentile position within the tissue to facilitate visualization across tissues and gene expression data types.

### Analysis of enhancer sequence conservation

phastCon conservation scores for human enhancers were downloaded from https://hgdownload.cse.ucsc.edu/goldenPath/hg38/phastCons20way/ and for the mouse enhancers https://hgdownload.cse.ucsc.edu/goldenpath/mm10/phastCons60way/. As a control for comparison to our lineage-specific enhancer groups, we used the ENCODE CRE annotations for all annotated enhancer elements within the human hg38 https://hgdownload.soe.ucsc.edu/gbdb/hg38/encode3/ccre/encodeCcreCombined.bb and mouse mm10 https://hgdownload.soe.ucsc.edu/gbdb/mm10/encode3/ccre/encodeCcreCombined.bb genomes, respectively. ENCODE CRE tracks were subsets for those annotated as “enhD|enhP”. The conservation score for each base position across a 1 kb probe centered on the enhancer was averaged to give a score for each enhancer instance.

### Identification of priming-altering SNPs within human iPSCs

ATAC-seq peaks were called separately for each replicate by MACS2 [91] (options: -q 0.01 –nomodel –keep-dup all); high confidence peaks were called as peaks present within at least two of the three replicates. SNPs and indels within the 10 HipSci donors were called using GATK [92]. BEDtools intersect was used to identify 7pcw late-embryonic brain enhancers which overlapped SNPs or ATAC peaks in any donor. High-confidence accessible enhancers were called as overlapping ATAC peaks in at least two of the three replicates for each donor line. Known motifs were called in these enhancers using Homer v4.11. Enrichment for TF motifs within enhancers coinciding with altered accessibility when an SNP is present was determined using Fisher's exact test. For the visualization of ATAC signal at interesting sites, deeptools v3.5.1 was used [93]. Donors were grouped into their genotypes on a per SNP basis, and then counts within the enhancer of interest were extracted and compressed to generate one bigwig per genotype using bedGraphToBigWig [93, 94]. These bigwigs were input into deeptools computeMatrix and plotProfile with mean and standard deviation plotted.

For visualization of Cut&Tag signal across HipSci donor lines, an initial quality check of paired-end reads was performed by FastQC v0.11.5 [95]. Nextera Transposase adapter and low-quality bases were eliminated using Cutadapt v1.17 [96]. Reads were mapped to the hg38 reference using bowtie2 v2.3.4.2; mapped pairs with mapping quality < 30 were removed. Duplicates were removed using GATK Picard MarkDuplicates. Genome browser tracks in bigwig format were generated using deepTools bamCoverage (options: binsize = 10, RPGC normalization).

## Supplementary Information

Additional file 1: Legends for Supplementary Figures and Tables. Fig. S1: Schematics of the histone modification signatures associated with various enhancer activity states. Fig. S2: Examples of Primed, Poised, and Non-primed hNPC enhancers. Fig. S3: Relative proportions of specific and non-specific lineage enhancer states. Fig. S4: Comparisons between ePrimed and ePoised human lineage-specific enhancer subgroups. Fig. S5: hESC H3K4me1 levels correlate with hESC H2K27ac levels at ePrimed enhancers. Fig. S6: Identification of mEND enhancer subgroups. Fig. S7: Genes associated with mECT and mEND enhancers. Fig. S8: Fetal and Somatic expression of ePrimed enhancer associated genes. Fig. S9: E11.5 mForebrain enhancers overlapping VISTA annotated enhancers. Fig. S10: Additional epigenetic profiling of fetal brain enhancers and DNA methylation of ePrimed enhancers within DNMT/TET KOs and gastrulation. Fig. S11: ePrimed enhancer associated gene network analysis. Fig. S12: Identification of human late-embryonic liver and kidney ePrimed enhancers. Fig. S13: Identification of mouse E11.5 liver and heart ePrimed enhancers. Fig. S14: Conservation of mouse and human ePrimed enhancers and associated genes. Fig. S15: Dynamics of ePrimed upon a cell fate transition into an alternative lineage. Fig. S16: DNA hypomethylation of ePrimed enhancers within somatic tissues. Fig. S17: ePrimed 7pcw fetal brain enhancers within HipSci donor lines.

Additional file 2: Table S1. Human ePrimed and eNon-primed enhancer annotations.

Additional file 3: Table S2. Mouse ePrimed and eNon-primed enhancer annotations.

Additional file 4: Table S3. Accession codes for datasets used.

## Data Availability

Human preimplantation scNMT-seq data generated in this study are available from the NCBI Gene Expression Omnibus repository under the accession number GSE279857 [97], Human iPSC CUT&Tag are available under the accession number GSE290517 [98], and Human iPSC ATAC-seq under the accession number GSE290515 [99]. Scripts used for data analysis are available from Github (https://github.com/DrChrisTodd/Enhancer_priming/) together with a Zenodo repository (https://zenodo.org/doi/10.5281/zenodo.15533417) [100].
