# Author Correction: Epigenetic priming of mammalian embryonic enhancer elements coordinates developmental gene networks

**DOI:** 10.1186/s13059-026-04290-w

**Published:** 2026-09-24

**Authors:** Christopher D. Todd, Jannat Ijaz, Fereshteh Torabi, Oleksandr Dovgusha, Stephen Bevan, Olivia Cracknell, Tim Lohoff, Stephen Clark, Ricard Argelaguet, Juliette Pearce, Ioannis Kafetzopoulos, Alice Santambrogio, Jennifer Nichols, Ferdinand von Meyenn, Ufuk Günesdogan, Stefan Schoenfelder, Wolf Reik

**Affiliations:** 1 Epigenetics Programme, Babraham Institute, Babraham Research Campus, Cambridge, CB22 3AT UK; 2 Göttingen Center for Molecular Biology, Department of Developmental Biology, University of Göttingen, Justus-Von-Liebig Weg 11, Göttingen, 37077 Germany; 3 Wellcome-MRC Cambridge Stem Cell Institute, Jeffrey Cheah Biomedical Centre, University of Cambridge, Puddicombe Way, Cambridge, CB2 0AW UK; 4 Present Address: Altos Labs, Cambridge Institute of Science, Granta Park, Cambridge, CB21 6GQ UK; 5 Present Address: Wellcome Sanger Institute, Wellcome Genome Campus, Hinxton, Cambridge, CB10 1SA UK; 6 Present Address: GSK, Gunnels Wood Road, Stevenage, SG1 2NY UK; 7 Present Address: Forbion, Gaertnerplatz 6, Munich, Germany; 8 Present Address: Institute for Cell and Tumor Biology, RWTH Aachen University Hospital, Aachen, Germany; 9 Present Address: Institute of Genetics and Cancer, The University of Edinburgh, Western General Hospital, Crewe Road, Edinburgh, EH4 2XU UK; 10 Present Address: Laboratory of Nutrition and Metabolic Epigenetics, Department of Health Sciences and Technology, ETH Zurich, Zurich, Switzerland; 11 Department of Medical and Molecular Genetics, Kings College London, London, UK

**Author Correction: Genome Biol 26, 214 (2025)**

**https://doi.org/10.1186/s13059-025–03658-8**

Following publication of the original article [1], the authors identified an error in the author name of Juliette Pearce.

The incorrect author name is: Juliette Pierce

The correct author name is: Juliette Pearce

The author group has been updated above and the original article [1] has been corrected.
