# Supplementary material for: Epigenetic priming of mammalian embryonic enhancer elements coordinates developmental gene networks
Source: Genome Biol. 2025 Jul 18;26:214. doi: 10.1186/s13059-025-03658-8 (PMC12272991; doi:10.1186/s13059-025-03658-8)
Supplement: Supplementary file 1 — Additional file 1: Legends for Supplementary Figures and Tables. Fig. S1: Schematics of the histone modification signatures associated with various enhancer activity states. Fig. S2: Examples of Primed, Poised, and Non-primed hNPC enhancers. Fig. S3: Relative proportions of specific and non-specific lineage enhancer states. Fig. S4: Comparisons between ePrimed and ePoised human lineage-specific enhancer subgroups. Fig. S5: hESC H3K4me1 levels correlate with hESC H2K27ac levels at ePrimed enhancers. Fig. S6: Identification of mEND enhancer subgroups. Fig. S7: Genes associated with mECT and mEND enhancers. Fig. S8: Fetal and Somatic expression of ePrimed enhancer associated genes. Fig. S9: E11.5 mForebrain enhancers overlapping VISTA annotated enhancers. Fig. S10: Additional epigenetic profiling of fetal brain enhancers and DNA methylation of ePrimed enhancers within DNMT/TET KOs and gastrulation. Fig. S11: ePrimed enhancer associated gene network analysis. Fig. S12: Identification of human late-embryonic liver and kidney ePrimed enhancers. Fig. S13: Identification of mouse E11.5 liver and heart ePrimed enhancers. Fig. S14: Conservation of mouse and human ePrimed enhancers and associated genes. Fig. S15: Dynamics of ePrimed upon a cell fate transition into an alternative lineage. Fig. S16: DNA hypomethylation of ePrimed enhancers within somatic tissues. Fig. S17: ePrimed 7pcw fetal brain enhancers within HipSci donor lines. [file 13059_2025_3658_MOESM1_ESM.pdf]

Fig. S1: Schematics of the histone modification signatures associated with various enhancer activity states

Active (H3K27ac+/H3K4me1+/H3K27me3-), Poised (H3K27ac-/H3K4me1+/H3K27me3+), Primed (H3K27ac-/H3K4me1+/H3K27me3-), or Inactive (H3K27ac-/H3K4me1-/H3K27me3-).

Fig. S2: Examples of Primed, Poised, and Non-primed hNPC enhancers.

Genome browser visualization examples of hNPC enhancers that were identified as being either **(A)** Primed, **(B)** Poised, or **(C)** Non-primed within hESCs. Each window represents a single enhancer instance with the enhancer annotated in red.

Fig. S3: Relative proportions of specific and non-specific lineage enhancer states.

Alluvial plots of relative proportions of lineage-specific enhancers (H3K27ac signal in a single cell lineage) and nonspecific (H3K27ac signal at enhancer within multiple cell lineages) and their respective epigenetic state within hESC/mESC cultures. Plots for all identified **(A)** hNPC enhancers, **(B)** hME enhancers, **(C)** E7.5 mECT enhancers, and **(D)** E7.5 mMES enhancers are shown.

Fig. S4: Comparisons between ePrimed and ePoised human lineage-specific enhancer subgroups.

(A) Heatmaps of ChIP-seq H3K27ac, H3K4me1 and H3K27me3 data within hESC, hNPC and hME cells in culture at enhancer elements with lineage-specific active states in hESC, hNPC or hME cells. Heatmaps are ordered by H3K4me1 signal at the enhancer within hESCs. **(B&C)** DNA methylation levels over 500bp core of active hESC enhancers (white) and hNPC, hME, 7pcw. brain, late-embryonic liver and kidney called enhancer sub-groups: ePrimed (ep.), eNon-primed (enp.), and ePoised (poi.) **(B)** by WGBS within in vitro primed hESC and **(C)** by scNMT-seq of in vivo human preimplantation embryos. hNPC poi. subgroup had insufficient coverage for quantification within the scNMT-seq data. Global methylation levels are displayed (red dashed line) along with results of ANOVA test between ePrimed and eNon-primed groups ($p<0.001$ ***, $p<0.05$ *). ANOVA tests were not performed for hNPC/hME ePoised groups due to insufficient number of elements for meaningful results.

Fig. S5: hESC H3K4me1 levels correlate with hESC H2K27ac levels at ePrimed enhancers

(A-D) ChIP-seq counts of lineage-specific primed (blue), non-primed (green) and intermediate (grey) sub-groups. Each dot is one enhancer element with a read count shown as log(RPKM) of 1.5kb window centred on the enhancer. Regression line of all lineage-specific enhancers within the group is shown with 95% confidence interval. Results of Pearson correlation coefficient test are also shown. Density plots of distribution of primed (blue) and non-primed (green) populations are shown on the x and y axes. **(A)** Read counts of hESC H3K4me1 and hNPC H3K27ac at hNPC enhancers. **(B)** Read counts of hESC H3K4me1 and hESC H3K27ac at hNPC enhancers. **(C)** Read counts of mESC H3K4me1 and mEND H3K27ac at E7.5 mEND enhancers. **(D)** Read counts of mESC H3K4me1 and mESC H3K27ac at E7.5 mEND enhancers.

Fig. S6: Identification of mEND enhancer subgroups.

(A) Heatmaps of ChIP-seq H3K27ac, H3K4me1 and H3K27me3 data within mESCs, and of H3K27ac within in vivo dissections of mECT, mEND, and mMES tissues for E7.5 embryos. Shown here are enhancer elements with lineage-specific active states in mESC cells or E7.5 mEND tissue. Enhancer groups called as ePrimed (high H3K4me1, low H3K27ac), eNon-primed (low H3K4me1, low H3K27ac), or Intermediate (intermediate H3K4me1) are displayed. **(B)** ATAC-seq profile for E7.5 mEND called enhancer groups within in vitro mESCs. Running averages in 50-bp windows around the centre of the enhancer (2 kb upstream and downstream) are shown alongside the averaged global signal (black dashed line). **(C-D)** DNA methylation levels over 500bp core of active mESC enhancers (white) and mEND called enhancer sub-groups: ePrimed (ep.) and eNon-primed (enp.) **(C)** by WGBS within in vitro naive mESC/mEpiLCs and **(D)** by scNMT-seq of in vivo mouse embryos at days E4.5 and E6.5. **(C-D)** Box plots show median levels and the first and third quartile, whiskers show 1.5x the interquartile range. Global methylation levels are displayed (red dashed line) along with results of ANOVA test between primed and non-primed groups ($p<0.001$ ***).

Fig. S7: Genes associated with mECT and mEND enhancers.

(A-B) Gene ontology enrichment analysis for **(A)** ePrimed and eNon-primed E7.5 mECT enhancer-associated genes, and **(B)** ePrimed and eNon-primed E7.5 mEND enhancer-associated genes. Significance threshold of $p<0.05$ is displayed (red dashed line). **(C)** Overlaid box and violin plots show

expression of enhancer-associated genes. Box plots show median levels and the first and third quartile, whiskers show 1.5× the interquartile range. Results from Welch two-sample t-test against E6.5 epiblast are displayed. Expression (log normalized counts) within in vivo scNMT-seq data collected from E6.5 epiblast and E7.5 ectoderm/ endoderm/ mesoderm tissues of genes exclusively associated with either ePrimed E7.5 mECT (yellow) enhancers, or eNon-primed E7.5 mECT (grey) enhancers. Median expression for each gene group within E6.5 epiblast is also shown (grey dashed line).

Fig. S8: Fetal and Somatic expression of ePrimed enhancer associated genes.

(A-D) Relative expression of enhancer-associated genes as defined by PCHiC/proximity hybrid model. Box plots show median levels and the first and third quartile, whiskers show 1.5x the interquartile range. Results from Wilcoxon signed-rank test between primed and non-primed enhancer sub-groups are displayed ($p<0.01$ **, $p<0.05$ *). **(A-B)** Relative expression within mouse E11.5 late-embryonic and somatic tissues (2 month old), of mECT enhancer-associated genes **(A)** and of mEND enhancer-associated genes **(B)**. **(C-D)** Relative expression within human late-embryonic tissues (72-129 post conception days) and somatic tissues (50-70 years of age), of hNPC enhancer-associated genes **(C)** and of hME enhancer-associated genes **(D)**.

Fig. S9: E11.5 mForebrain enhancers overlapping VISTA annotated enhancers.

(A) Relative percentage of VISTA browser enhancers overlapping called E11.5 forebrain enhancers which are annotated for activity within respective embryonic tissues. VISTA enhancers overlapping all E11.5 forebrain enhancers are annotated in red, VISTA enhancers overlapping lineage-specific E11.5 forebrain enhancers are annotated in blue. **(B)** Representative images of reporter assay performed within E11.5 embryos for VISTA browser enhancers overlapping lineage-specific E11.5 forebrain enhancers. Tissues annotated with enhancer activity are listed below.

Fig. S10: Additional epigenetic profiling of fetal brain enhancers and DNA methylation of ePrimed enhancers within DNMT/TET KOs and gastrulation.

(A) ATAC-seq profile for 7pcw late-embryonic brain called enhancer groups within in vitro hESCs. Running averages in 50-bp windows around the centre of the enhancer (2 kb upstream and

downstream) are shown alongside the averaged global signal (black dashed line). **(B)** DNA methylation levels by WGBS within in vitro primed hESC over 500bp core of active hESC enhancers (white) and 7pcw late-embryonic brain called enhancer sub-groups: ePrimed (ep.) and eNon-primed (enp.) **(C)** ATAC-seq profile for E11.5 forebrain called enhancer groups within in vitro mESCs. Running averages in 50-bp windows around the centre of the enhancer (2 kb upstream and downstream) are shown alongside the averaged global signal (black dashed line). **(D)** DNA methylation levels by WGBS within in vitro mESC over 500bp core of active mESC enhancers (white) and E11.5 forebrain called enhancer sub-groups: ePrimed (ep.) and eNon-primed (enp.). Global DNA methylation shown by red dashed line. **(E)** DNA methylation levels by WGBS within in vitro primed hESC with combinations of knockouts performed for DNMT and TET genes over 500bp core of active hESC enhancers (white) and hNPC and hME called enhancer sub-groups: ePrimed (ep.) and eNon-primed (enp.). Global DNA methylation shown by red dashed line. Knockout lines shown are wild type control (WT), DNMT3A/3B double knockout (DNMT DKO), TET1-3 triple knockout (TET TKO), DNMT3A/3B and TET1-3 pentuple knockout (DNMT & TET PKO), PKO line with TET1 rescue, and PKO line with TET3 rescue. **(F)** DNA methylation levels by scNMT-seq of in vivo mouse embryos at days E6.5 and E7.5 over 500bp core of E7.5 mECT, E7.5 mMES, E7.5 mEND, and E11.5 forebrain called enhancer sub-groups: eNon-primed (enp.) and ePrimed (ep.). Global methylation of all cells within the group are shown in white. **(B,D-F)** Box plots show median levels and the first and third quartile, whiskers show 1.5x the interquartile range. Results of ANOVA test between indicated groups are shown ($p<0.001$ ***).

Fig. S11: ePrimed enhancer associated gene network analysis.

(A-C) STRING database inferred networks of ePrimed enhancer associated transcription factors. **(A)** hNPC upregulated TFs associated with hNPC ePrimed enhancers. **(B)** Late-embryonic liver expressed TFs associated with late-embryonic liver ePrimed enhancers. **(C)** Late-embryonic brain expressed TFs associated with 7pcw. brain ePrimed enhancers. **(A-C)** Gene nodes are coloured to highlight genes within enriched Gene ontologies with colour legend shown. Legend for protein-protein interaction types is also shown. **(D)** Venn diagrams showing the overlap between all TFs associated with mouse and human respective ePrimed enhancer groups.

Fig. S12: Identification of human late-embryonic liver and kidney ePrimed enhancers.

(A) Heatmaps of ChIP-seq H3K27ac, H3K4me1 and H3K27me3 data within hESC, hNPC and hME cells in culture, and H3K27ac of late-embryonic Brain, Liver and Kidney tissues at enhancer elements with lineage-specific active states in late-embryonic Liver and Kidney tissues alongside those active within hESCs. Heatmaps are ordered by H3K4me1 signal at the enhancer within hESCs. Enhancer groups called as ePrimed (high H3K4me1, low H3K27ac), eNon-primed (low H3K4me1, low H3K27ac), or Intermediate (intermediate H3K4me1) are displayed. **(B)** ATAC-seq profile for late-embryonic Liver and Kidney called enhancer groups within in vitro primed hESCs. Running averages in 50-bp windows around the centre of the enhancer (2 kb upstream and downstream) are shown alongside the averaged global signal (black dashed line). **(C&D)** DNA methylation levels over 500bp core of active hESC enhancers (white) and late-embryonic Liver and Kidney called enhancer sub-groups: ePrimed (ep.) and eNon-primed (enp.) **(C)** by WGBS within in vitro primed hESC and **(D)** by scNMT-seq of in vivo human preimplantation embryos. **(E,F)** Gene ontology enrichment analysis for ePrimed and eNon-primed enhancer-associated genes as defined by PCHiC/proximity hybrid model. Significance threshold of $p<0.05$ is displayed (red dashed line). **(E)** Analysis of ePrimed and eNon-primed late-embryonic liver enhancer-associated genes and **(F)** ePrimed and eNon-primed late-embryonic kidney enhancer-associated genes.

Fig. S13: Identification of mouse E11.5 liver and heart ePrimed enhancers.

(A) Heatmaps of ChIP-seq H3K27ac, H3K4me1 and H3K27me3 data within mESC, and H3K27ac data within in vivo dissections of mECT, mEND, and mMES tissues for E7.5 embryos and Forebrain, Liver and Heart tissues for E11.5 embryos. Heatmaps show signal at enhancer elements with lineage-specific active states in E11.5 Liver and Heart tissues, along with those active within mESCs. Heatmaps are ordered by H3K4me1 signal at the enhancer within mESCs. Enhancer groups called as ePrimed (high H3K4me1, low H3K27ac), eNon-primed (low H3K4me1, low H3K27ac), or Intermediate (intermediate H3K4me1) are displayed. **(B)** ATAC-seq profile for E11.5 Liver and Heart called enhancer groups within in vitro mESCs. Running averages in 50-bp windows around the centre of the enhancer (2 kb upstream and downstream) are shown alongside the averaged global signal (black dashed line). **(C&D)** DNA methylation levels over 500bp core of active mESC enhancers (white) and

E11.5 Liver and Heart called enhancer sub-groups: ePrimed (ep.) and eNon-primed (enp.) **(C)** by WGBS within in vitro mESCs and **(D)** by scNMT-seq of in vivo E4.5 and E6.5 embryos.

Fig. S14: Conservation of mouse and human ePrimed enhancers and associated genes.

(A) Number of conserved genes associated with 7pcw brain ePrimed enhancers and E11.5 mForebrain ePrimed enhancers. **(B)** Gene ontology enrichments of genes associated with both 7pcw brain and E11.5 mForebrain ePrimed enhancers. **(C)** Number of conserved genes associated with human late-embryonic liver ePrimed enhancers and mouse E11.5 liver ePrimed enhancers. **(D)** Gene ontology enrichments of genes associated with both human late-embryonic liver and mouse E11.5 liver ePrimed enhancers. **(E&F)** Sequence conservation score from phastCon model averaged over ePrimed (ep.), eNon-primed (enp.), ePoised (poi.) and all annotated ENCODE enhancers (all enh) for enhancers both within **(E)** human genome and **(F)** mouse genome.

Fig. S15: Dynamics of ePrimed upon a cell fate transition into an alternative lineage.

(A) DNA methylation levels over 500bp core of E7.5 mECT, E7.5 mEND, E7.5 mMES. E11.5 forebrain, E11.5 liver, and E11.5 heart called enhancer sub-groups: ePrimed (ep.) and eNon-primed (enp.) by scNMT-seq of in vivo mouse embryos at days E6.5 and E7.5. Global levels across the data is shown for each cell (white). **(B)** ChIP-seq signal intensity across a 1kb probe centered over hNPC, hME, 7pcw. brain, late-embryonic liver, and late-embryonic kidney called enhancer sub-groups: ePrimed (ep.), eNon-primed (enp.), and ePoised (poi.). **(A+B)** Active enhancer groups for each tissue are highlighted in green, and obsolete enhancer groups within each lineage are highlighted in red.

Fig. S16: DNA hypomethylation of ePrimed enhancers within somatic tissues.

DNA methylation levels by WGBS-seq of in vivo human somatic tissues (50-70 years of age) over 500bp core of hME, hNPC and 7pcw late-embryonic brain called enhancer sub-groups: eNon-primed (enp.) and ePrimed (ep.). Average methylation across all somatic tissues for each enhancer is also plotted. Box plots show median levels and the first and third quartile, whiskers show 1.5x the interquartile range. Global methylation calculated at 10kb running windows (white) are shown along with results of ANOVA test between indicated groups ($p<0.001$ ***).

Fig. S17: ePrimed 7pcw fetal brain enhancers within HipSci donor lines.

(A) Percentage of ePrimed and eNon-primed 7pcw late-embryonic brain enhancers that overlap ATAC peaks within at least 2 of the 3 replicates per HipSci donor line. **(B)** Genome browser visualisation of respective examples of ePrimed 7pcw enhancers within the HipSci donor lines. CUT&Tag signal for H3K4me1 (blue) and H3K27ac (green) are shown. The centre for each enhancer is annotated below (red).

Additional File 2: Table S1

Genomic coordinates for center points of annotated human ePrimed and eNon-primed enhancer subgroups. Reference genome hg38. Coordinates are in BED format with: "Chromosome", "Start", "End", "Class of Enhancers".

Additional File 3: Table S2

Genomic coordinates for center points of annotated mouse ePrimed and eNon-primed enhancer subgroups. Reference genome mm10. Coordinates are in BED format with: "Chromosome", "Start", "End", "Class of Enhancers".

Additional File 4: Table S3

Accession codes for datasets used in this manuscript, detailing the organism, cell/tissue type, data type, and Accession.

Fig. S1

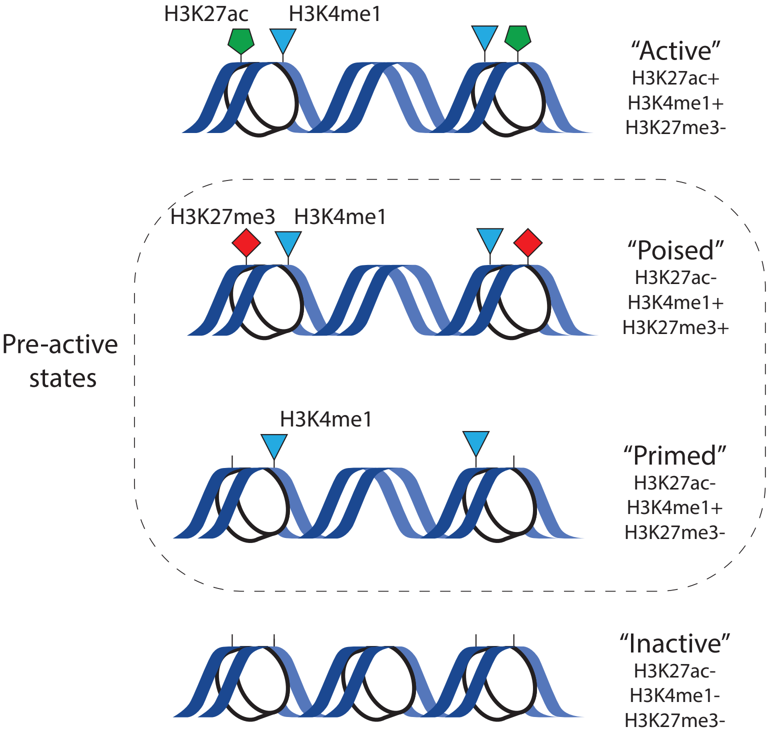

H3K27ac
H3K4me1
"Active"
H3K27ac+
H3K4me1+
H3K27me3-
H3K27me3
H3K4me1
"Poised"
H3K27ac-
H3K4me1+
H3K27me3+
Pre-active states
H3K4me1
"Primed"
H3K27ac-
H3K4me1+
H3K27me3-
"Inactive"
H3K27ac-
H3K4me1-
H3K27me3-

Fig. S2

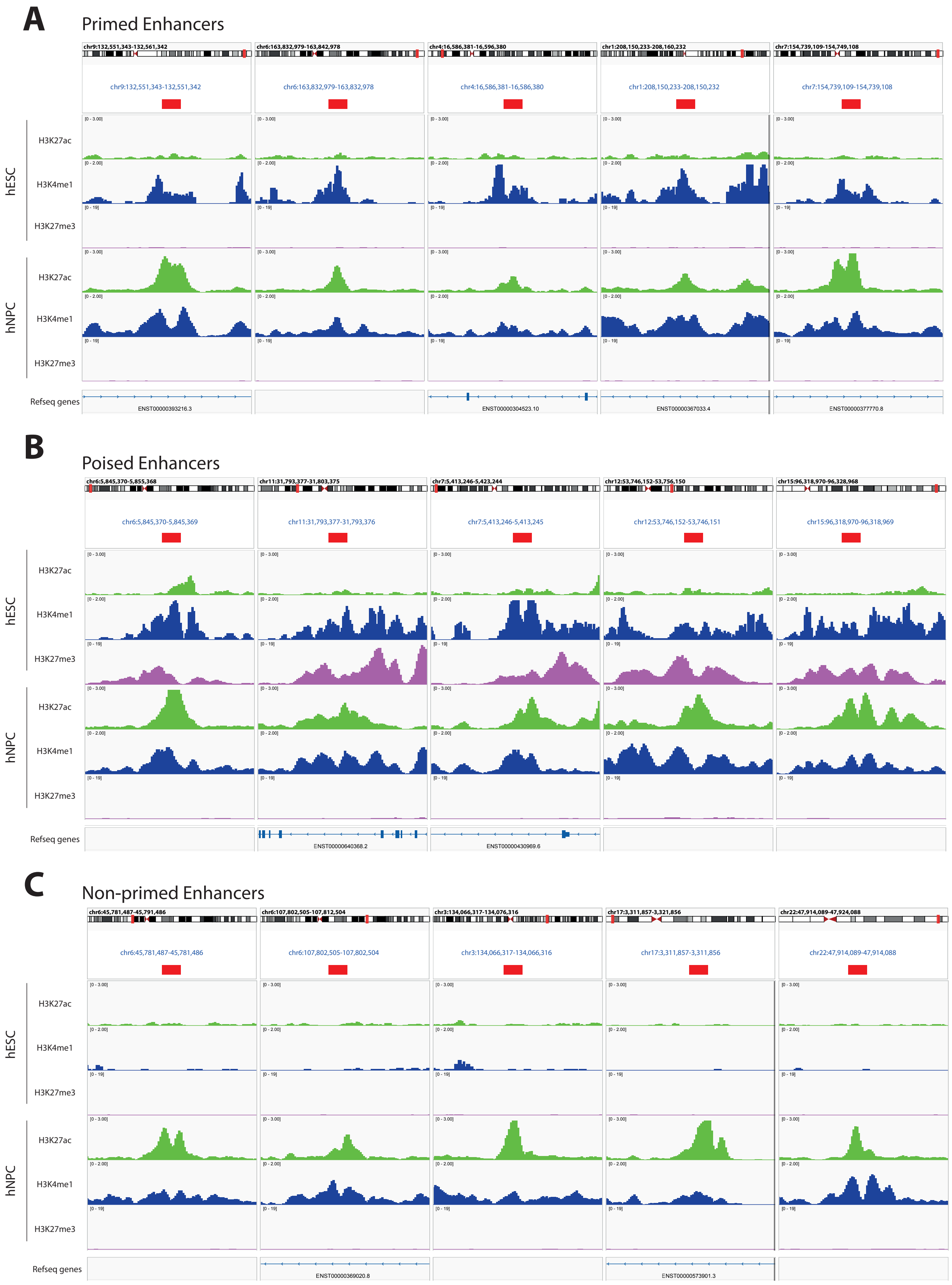
A
Primed Enhancers
chr9:132,551,343-132,561,342
chr6:163,832,979-163,842,978
chr4:16,586,381-16,596,380
chr1:208,150,233-208,160,232
chr7:154,739,109-154,749,108
chr9:132,551,343-132,551,342
chr6:163,832,979-163,832,978
chr4:16,586,381-16,586,380
chr1:208,150,233-208,150,232
chr7:154,739,109-154,739,108
hESC
hNPC
H3K27ac
H3K4me1
H3K27me3
Refseq genes
ENST00000393216.3
ENST00000304523.10
ENST00000367033.4
ENST00000377770.8
B
Poised Enhancers
chr6:5,845,370-5,855,368
chr11:31,793,377-31,803,375
chr7:5,413,246-5,423,244
chr12:53,746,152-53,756,150
chr15:96,318,970-96,328,968
chr6:5,845,370-5,845,369
chr11:31,793,377-31,793,376
chr7:5,413,246-5,413,245
chr12:53,746,152-53,746,151
chr15:96,318,970-96,318,969
ENST00000640368.2
ENST00000430969.6
C
Non-primed Enhancers
chr6:45,781,487-45,791,486
chr6:107,802,505-107,812,504
chr3:134,066,317-134,076,316
chr17:3,311,857-3,321,856
chr22:47,914,089-47,924,088
chr6:45,781,487-45,781,486
chr6:107,802,505-107,802,504
chr3:134,066,317-134,066,316
chr17:3,311,857-3,311,856
chr22:47,914,089-47,914,088
ENST00000369020.8
ENST00000573901.3

Fig. S3

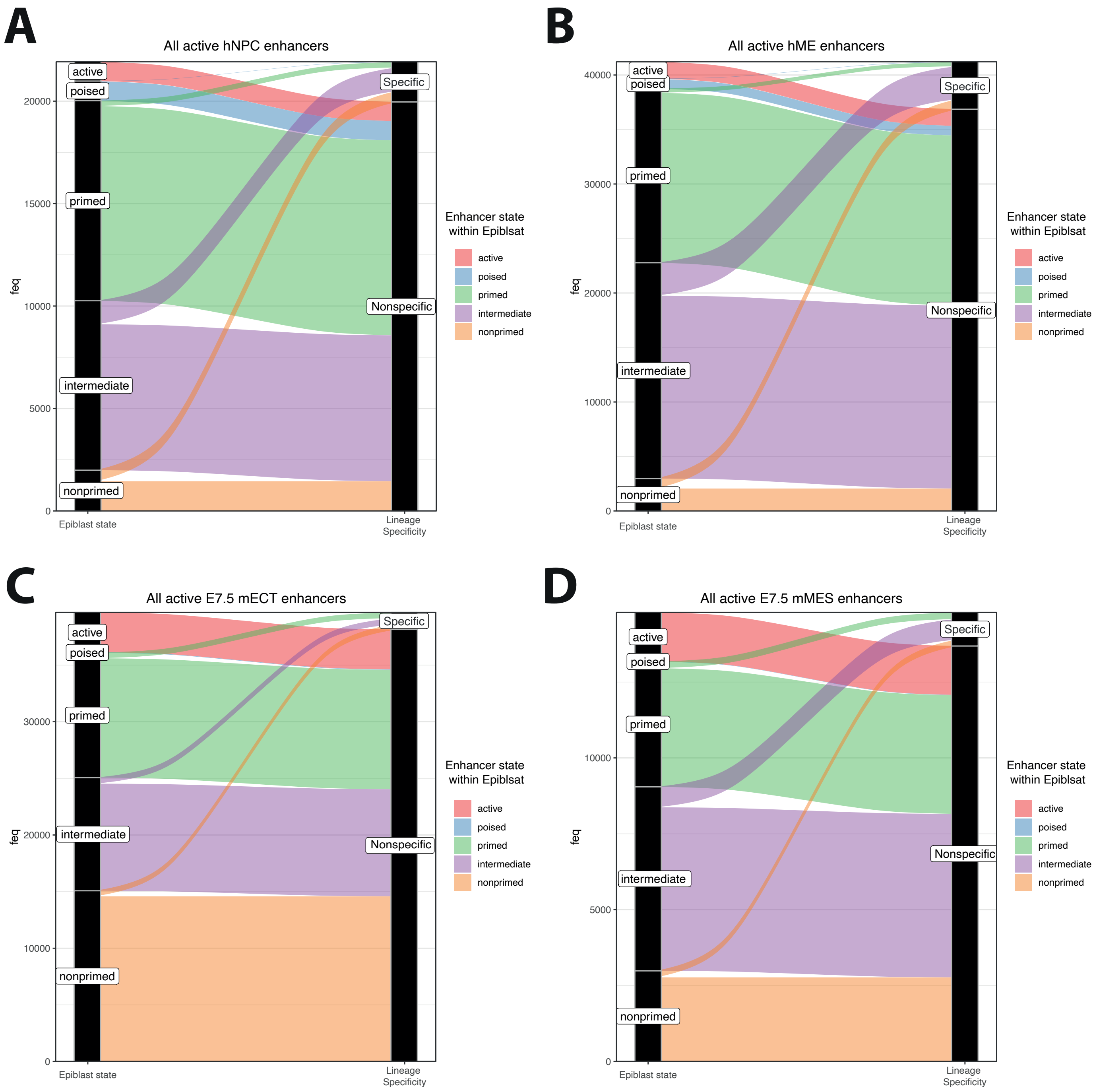

A
All active hNPC enhancers
B
All active hME enhancers
C
All active E7.5 mECT enhancers
D
All active E7.5 mMES enhancers
feq
active
poised
primed
intermediate
nonprimed
Specific
Nonspecific
Epiblast state
Lineage Specificity
Enhancer state within Epiblsat
active
poised
primed
intermediate
nonprimed
0
5000
10000
15000
20000
30000
40000

Fig. S4

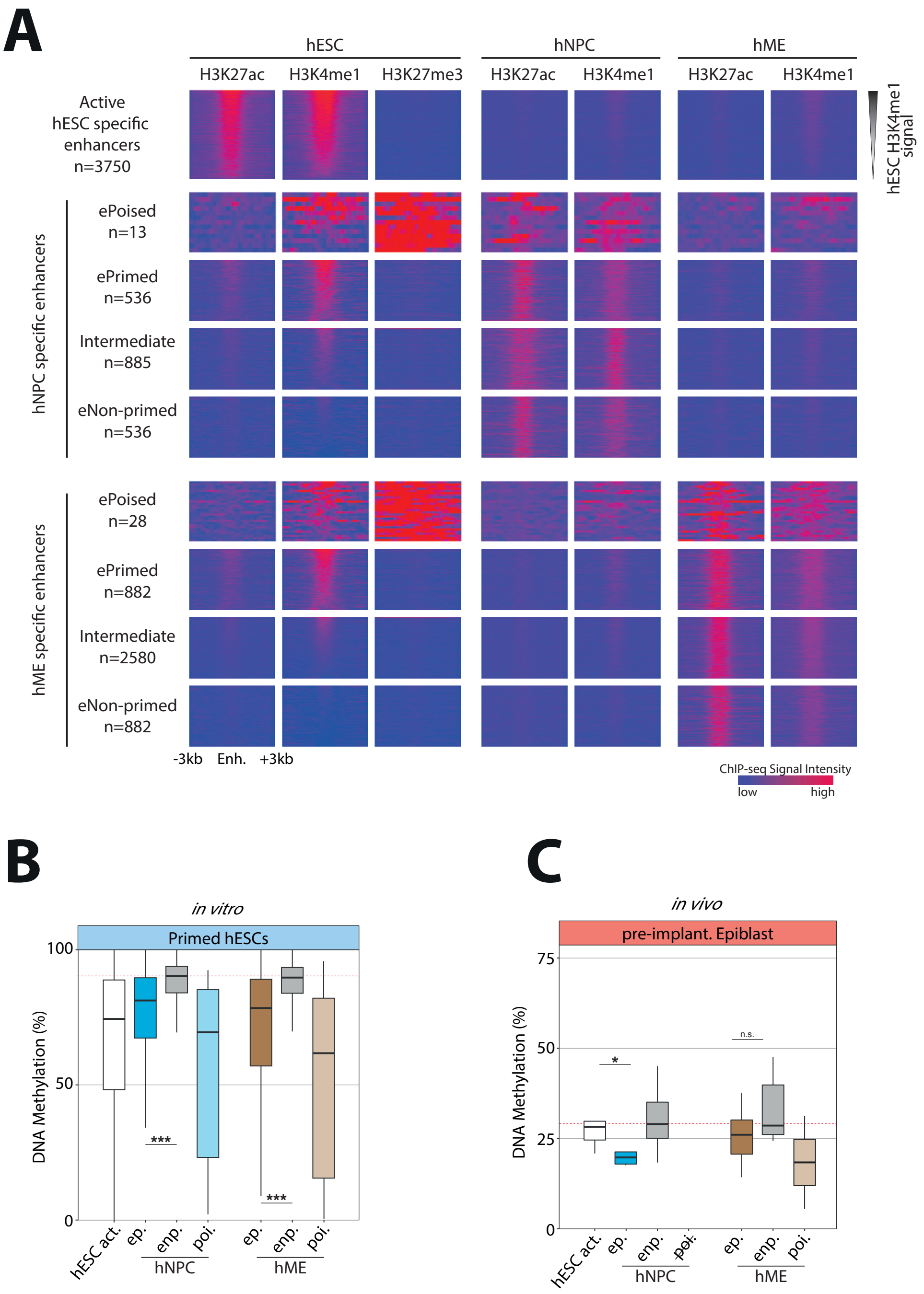
A
hESC
hNPC
hME
H3K27ac
H3K4me1
H3K27me3
Active hESC specific enhancers n=3750
hESC H3K4me1 signal
hNPC specific enhancers
ePoised n=13
ePrimed n=536
Intermediate n=885
eNon-primed n=536
hME specific enhancers
ePoised n=28
ePrimed n=882
Intermediate n=2580
eNon-primed n=882
-3kb
Enh.
+3kb
ChIP-seq Signal Intensity
low
high
B
in vitro
Primed hESCs
DNA Methylation (%)
100
50
0
***
***
hESC act.
ep.
enp.
poi.
hNPC
hME
C
in vivo
pre-implant. Epiblast
DNA Methylation (%)
75
50
25
0
*
n.s.
hESC act.
ep.
enp.
poi.
hNPC
hME

Fig. S5

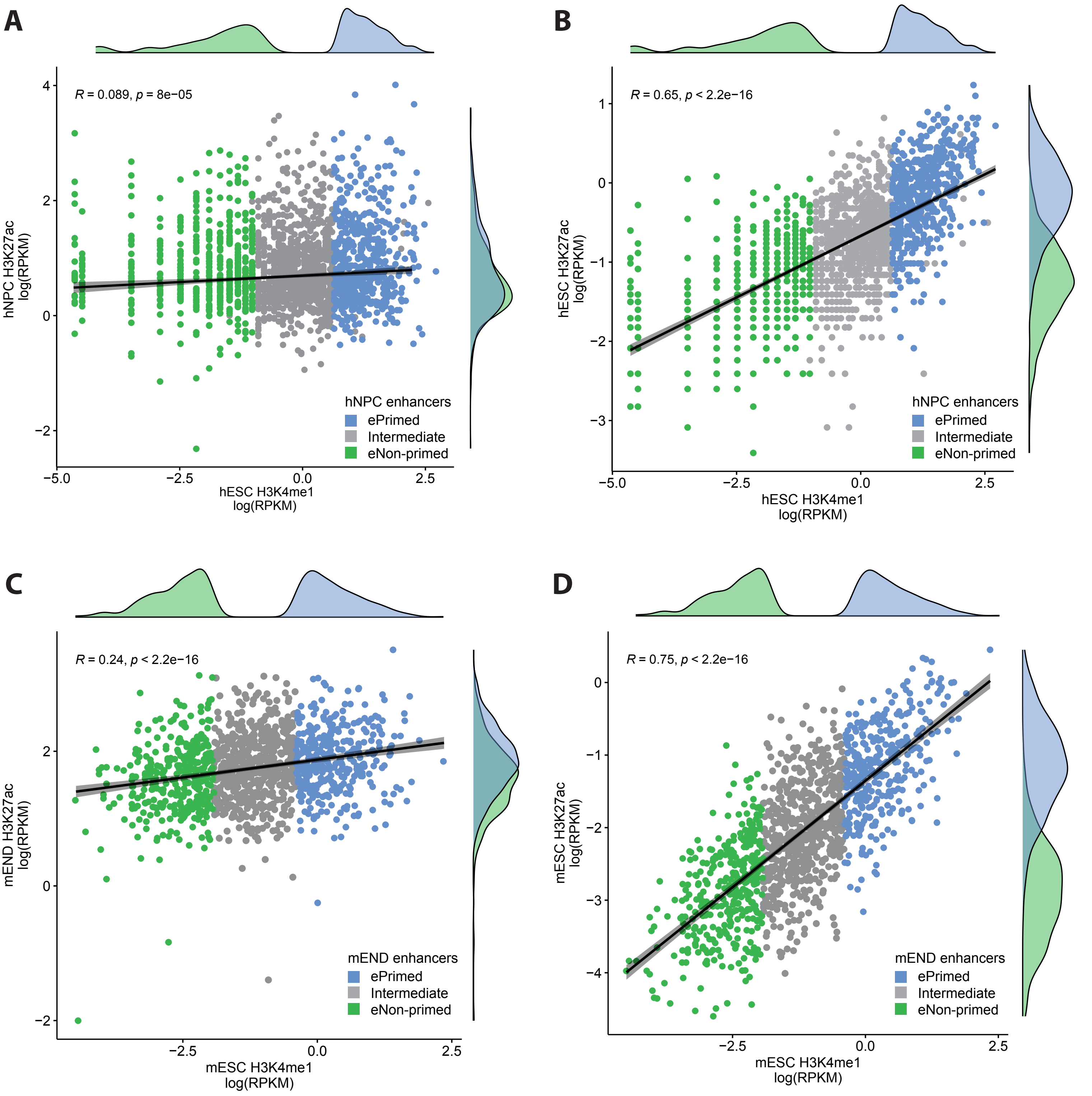
A
R = 0.089, p = 8e−05
hNPC H3K27ac log(RPKM)
hESC H3K4me1 log(RPKM)
hNPC enhancers
ePrimed
Intermediate
eNon-primed
B
R = 0.65, p < 2.2e−16
hESC H3K27ac log(RPKM)
hESC H3K4me1 log(RPKM)
hNPC enhancers
ePrimed
Intermediate
eNon-primed
C
R = 0.24, p < 2.2e−16
mEND H3K27ac log(RPKM)
mESC H3K4me1 log(RPKM)
mEND enhancers
ePrimed
Intermediate
eNon-primed
D
R = 0.75, p < 2.2e−16
mESC H3K27ac log(RPKM)
mESC H3K4me1 log(RPKM)
mEND enhancers
ePrimed
Intermediate
eNon-primed

Fig. S6

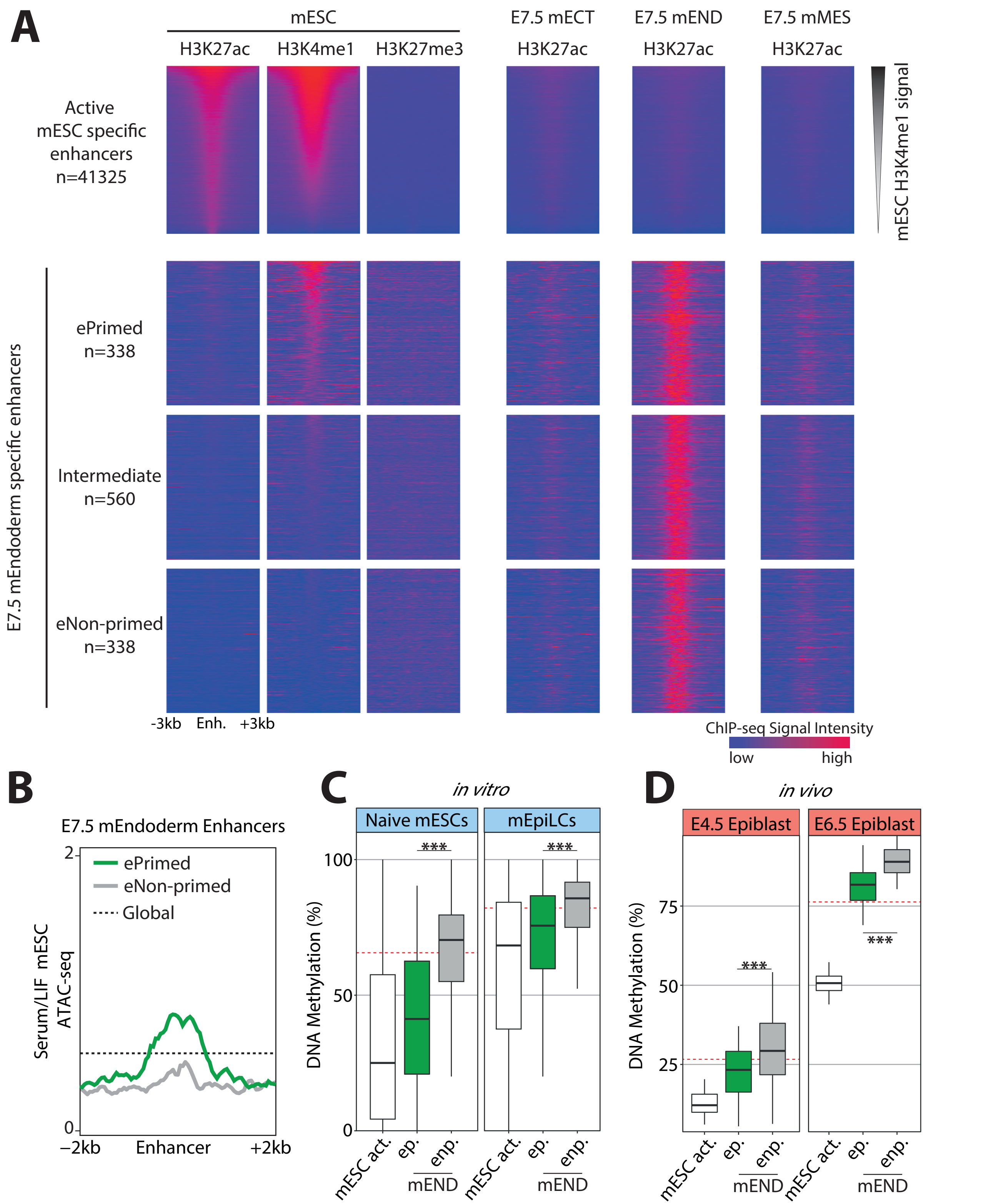

A
mESC
H3K27ac
H3K4me1
H3K27me3
E7.5 mECT
H3K27ac
E7.5 mEND
H3K27ac
E7.5 mMES
H3K27ac
Active mESC specific enhancers n=41325
mESC H3K4me1 signal
E7.5 mEndoderm specific enhancers
ePrimed n=338
Intermediate n=560
eNon-primed n=338
-3kb
Enh.
+3kb
ChIP-seq Signal Intensity
low
high
B
E7.5 mEndoderm Enhancers
Serum/LIF mESC ATAC-seq
ePrimed
eNon-primed
Global
2
0
−2kb
Enhancer
+2kb
C
in vitro
Naive mESCs
mEpiLCs
DNA Methylation (%)
100
50
0
***
***
mESC act.
ep.
enp.
mEND
D
in vivo
E4.5 Epiblast
E6.5 Epiblast
75
50
25
***
***

Fig. S7

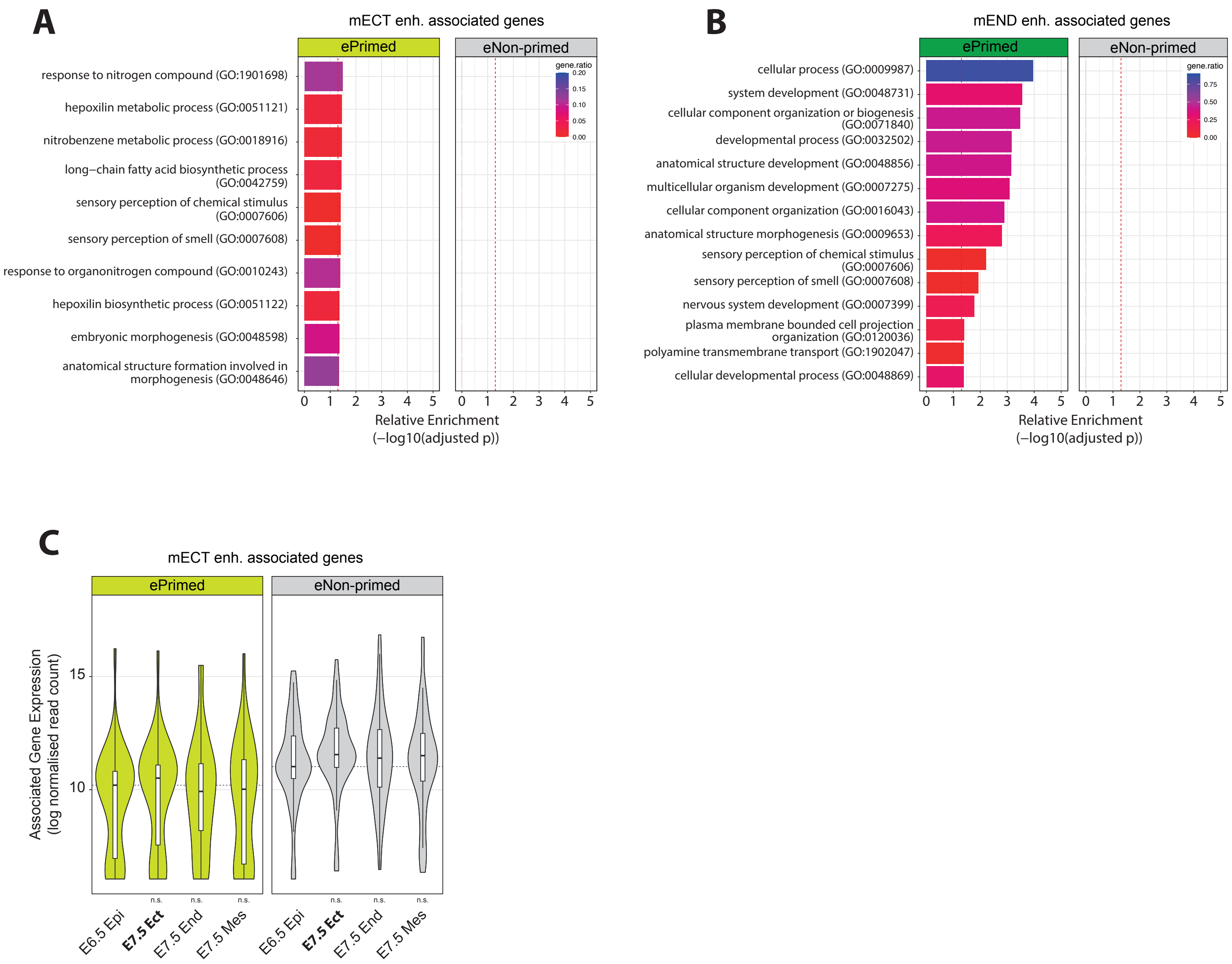
A
mECT enh. associated genes
ePrimed
eNon-primed
response to nitrogen compound (GO:1901698)
hepoxilin metabolic process (GO:0051121)
nitrobenzene metabolic process (GO:0018916)
long−chain fatty acid biosynthetic process (GO:0042759)
sensory perception of chemical stimulus (GO:0007606)
sensory perception of smell (GO:0007608)
response to organonitrogen compound (GO:0010243)
hepoxilin biosynthetic process (GO:0051122)
embryonic morphogenesis (GO:0048598)
anatomical structure formation involved in morphogenesis (GO:0048646)
gene.ratio
0.20
0.15
0.10
0.05
0.00
0 1 2 3 4 5
Relative Enrichment
(−log10(adjusted p))
B
mEND enh. associated genes
ePrimed
eNon-primed
cellular process (GO:0009987)
system development (GO:0048731)
cellular component organization or biogenesis (GO:0071840)
developmental process (GO:0032502)
anatomical structure development (GO:0048856)
multicellular organism development (GO:0007275)
cellular component organization (GO:0016043)
anatomical structure morphogenesis (GO:0009653)
sensory perception of chemical stimulus (GO:0007606)
sensory perception of smell (GO:0007608)
nervous system development (GO:0007399)
plasma membrane bounded cell projection organization (GO:0120036)
polyamine transmembrane transport (GO:1902047)
cellular developmental process (GO:0048869)
gene.ratio
0.75
0.50
0.25
0.00
0 1 2 3 4 5
Relative Enrichment
(−log10(adjusted p))
C
mECT enh. associated genes
ePrimed
eNon-primed
Associated Gene Expression
(log normalised read count)
15
10
n.s.
E6.5 Epi
E7.5 Ect
E7.5 End
E7.5 Mes

Fig. S8

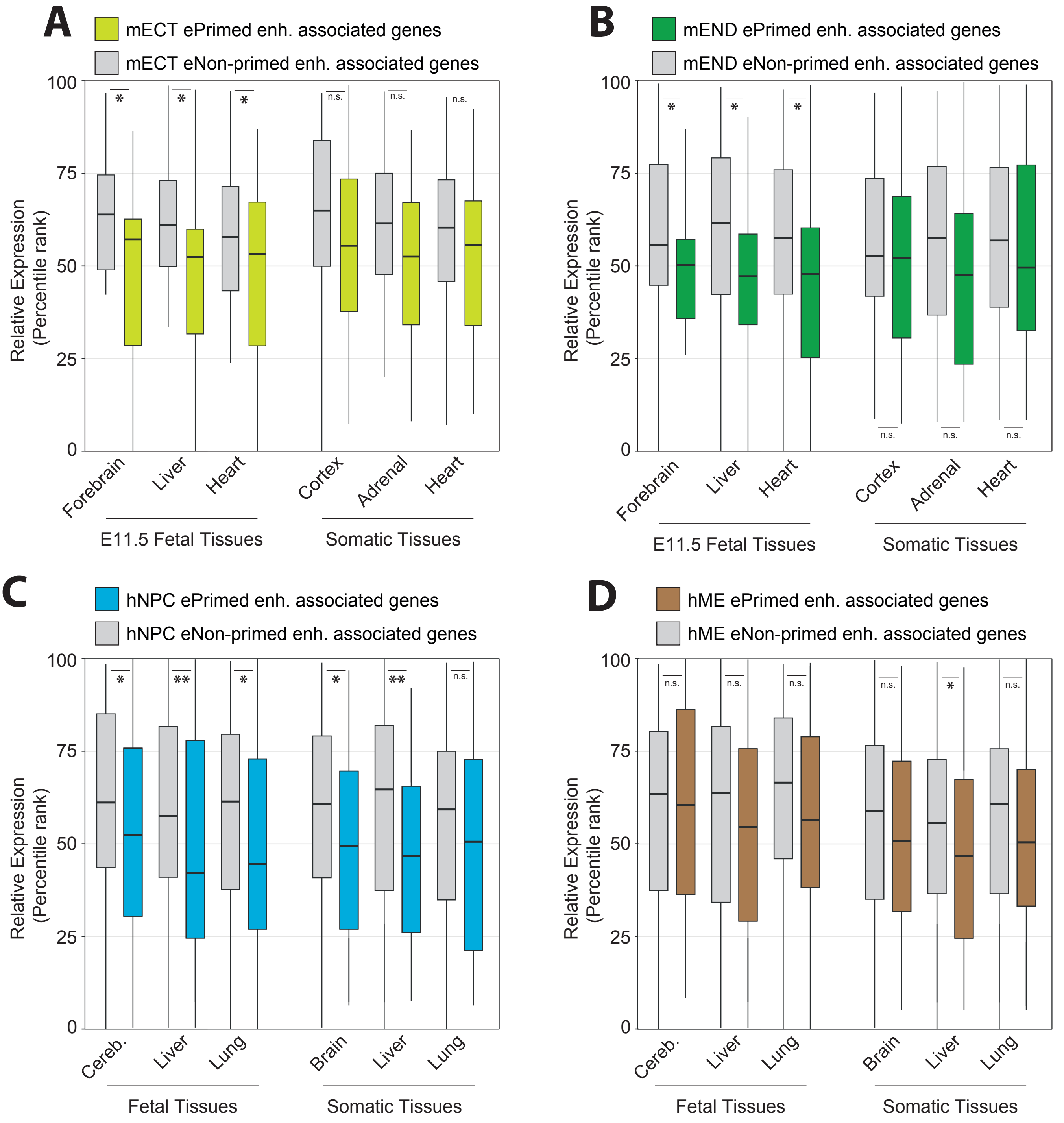
A
mECT ePrimed enh. associated genes
mECT eNon-primed enh. associated genes
Relative Expression (Percentile rank)
100
75
50
25
0
*
*
*
n.s.
n.s.
n.s.
Forebrain
Liver
Heart
Cortex
Adrenal
Heart
E11.5 Fetal Tissues
Somatic Tissues
B
mEND ePrimed enh. associated genes
mEND eNon-primed enh. associated genes
Relative Expression (Percentile rank)
*
*
*
n.s.
n.s.
n.s.
Forebrain
Liver
Heart
Cortex
Adrenal
Heart
E11.5 Fetal Tissues
Somatic Tissues
C
hNPC ePrimed enh. associated genes
hNPC eNon-primed enh. associated genes
Relative Expression (Percentile rank)
*
**
*
*
**
n.s.
Cereb.
Liver
Lung
Brain
Liver
Lung
Fetal Tissues
Somatic Tissues
D
hME ePrimed enh. associated genes
hME eNon-primed enh. associated genes
Relative Expression (Percentile rank)
n.s.
n.s.
n.s.
n.s.
*
n.s.
Cereb.
Liver
Lung
Brain
Liver
Lung
Fetal Tissues
Somatic Tissues

Fig. S9

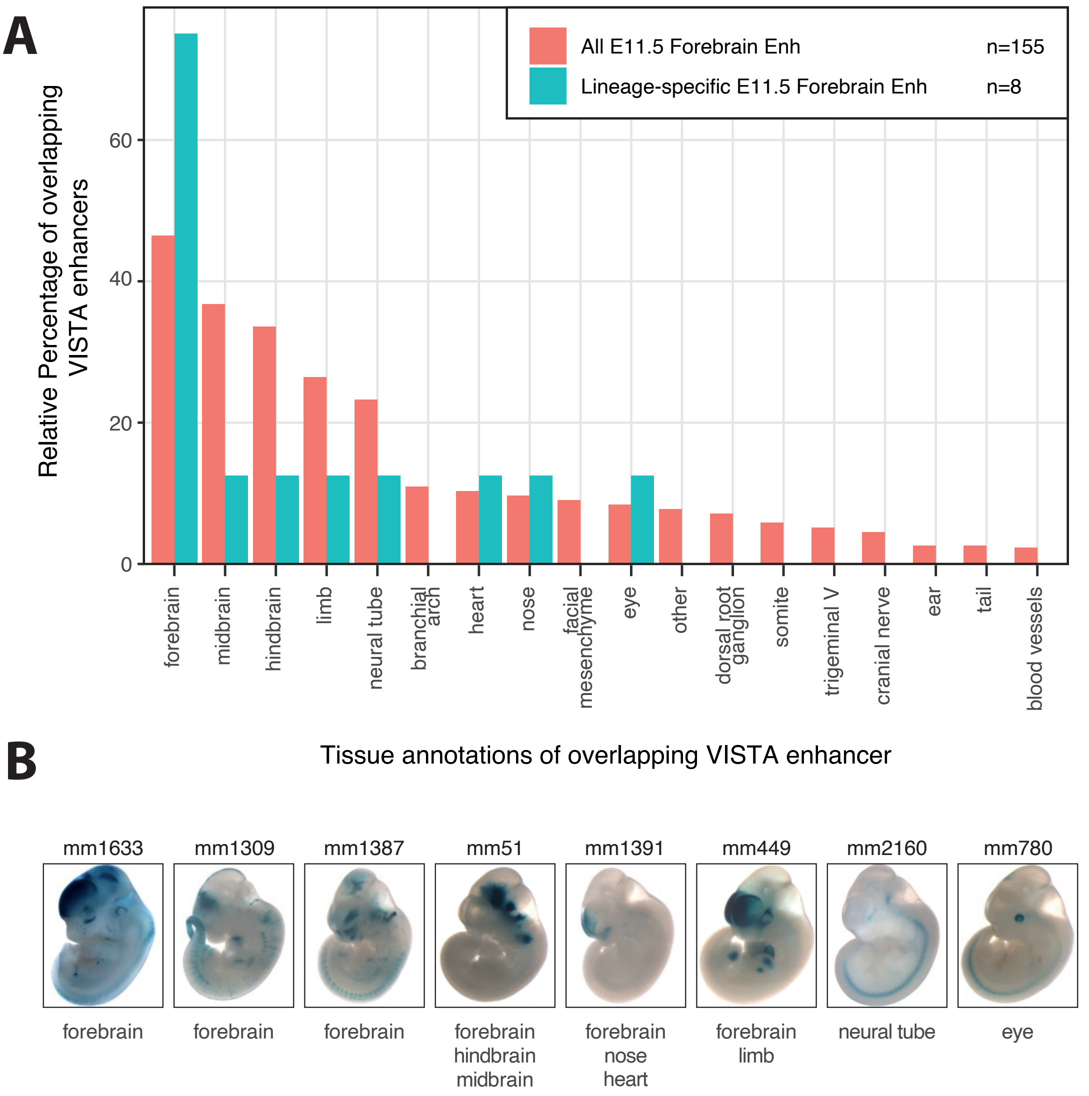
A
All E11.5 Forebrain Enh n=155
Lineage-specific E11.5 Forebrain Enh n=8
Relative Percentage of overlapping VISTA enhancers
0
20
40
60
forebrain
midbrain
hindbrain
limb
neural tube
branchial arch
heart
nose
facial mesenchyme
eye
other
dorsal root ganglion
somite
trigeminal V
cranial nerve
ear
tail
blood vessels
Tissue annotations of overlapping VISTA enhancer
B
mm1633
mm1309
mm1387
mm51
mm1391
mm449
mm2160
mm780
forebrain
forebrain
forebrain
forebrain hindbrain midbrain
forebrain nose heart
forebrain limb
neural tube
eye

Fig. S10

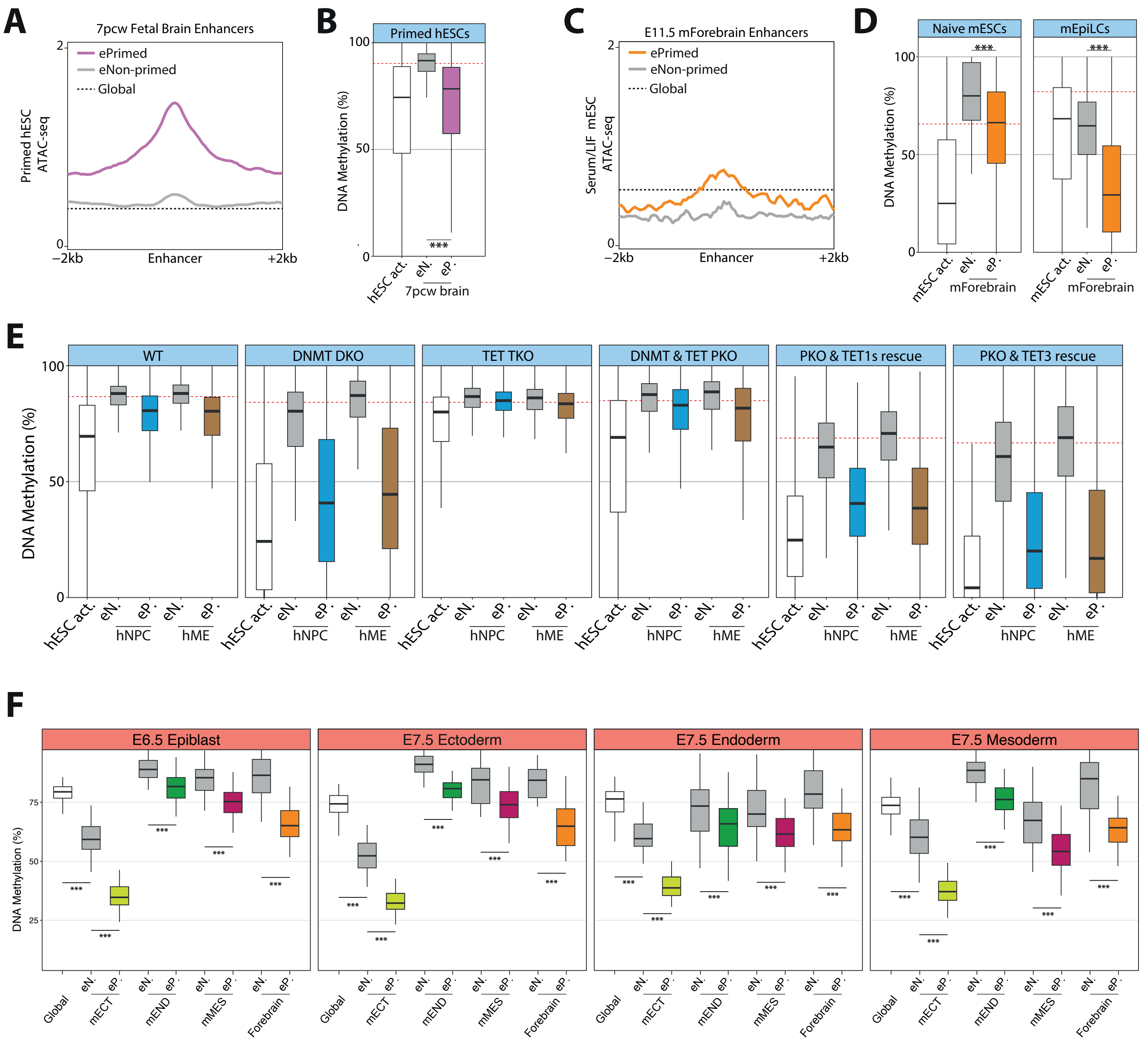

A
7pcw Fetal Brain Enhancers
ePrimed
eNon-primed
Global
Primed hESC ATAC-seq
–2kb
Enhancer
+2kb
B
Primed hESCs
DNA Methylation (%)
***
hESC act.
eN.
eP.
7pcw brain
C
E11.5 mForebrain Enhancers
ePrimed
eNon-primed
Global
Serum/LIF mESC ATAC-seq
–2kb
Enhancer
+2kb
D
Naive mESCs
mEpiLCs
***
***
DNA Methylation (%)
mESC act.
eN.
eP.
mForebrain
E
WT
DNMT DKO
TET TKO
DNMT & TET PKO
PKO & TET1s rescue
PKO & TET3 rescue
DNA Methylation (%)
hESC act.
eN.
eP.
hNPC
hME
F
E6.5 Epiblast
E7.5 Ectoderm
E7.5 Endoderm
E7.5 Mesoderm
DNA Methylation (%)
***
Global
eN.
eP.
mECT
mEND
mMES
Forebrain

Fig. S11

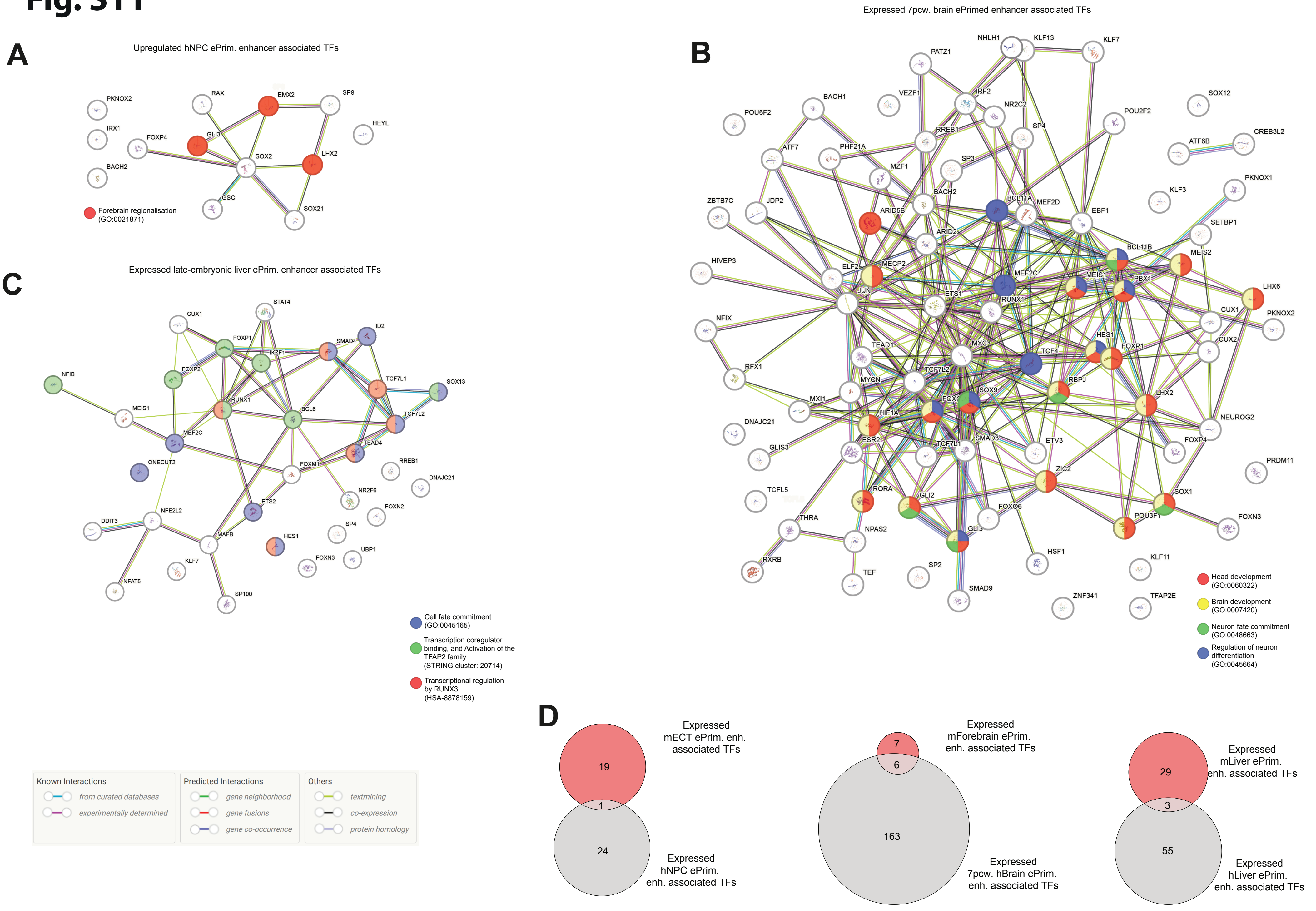

A
Upregulated hNPC ePrim. enhancer associated TFs
PKNOX2
RAX
EMX2
SP8
HEYL
IRX1
FOXP4
GLI3
SOX2
LHX2
BACH2
GSC
SOX21
Forebrain regionalisation (GO:0021871)
B
Expressed 7pcw. brain ePrimed enhancer associated TFs
NHLH1
KLF13
KLF7
PATZ1
BACH1
VEZF1
IRF2
NR2C2
SOX12
POU6F2
RREB1
SP4
POU2F2
ATF6B
CREB3L2
ATF7
PHF21A
SP3
MZF1
KLF3
PKNOX1
ZBTB7C
JDP2
BACH2
ARID5B
BCL11A
MEF2D
EBF1
SETBP1
ARID2
HIVEP3
ELF2
MECP2
BCL11B
MEIS2
MEF2C
MEIS1
PBX1
LHX6
JUN
ETS1
RUNX1
NFIX
CUX1
PKNOX2
CUX2
HES1
TEAD1
MYC
TCF4
FOXP1
RFX1
TCF7L2
MYCN
RBPJ
SOX9
MXI1
LHX2
NEUROG2
HIF1A
DNAJC21
ESR2
SMAD3
TCF7L1
ETV3
FOXP4
GLIS3
PRDM11
ZIC2
TCFL5
RORA
GLI2
SOX1
FOXO6
THRA
POU3F1
FOXN3
GLI3
NPAS2
RXRB
HSF1
KLF11
SP2
TEF
SMAD9
ZNF341
TFAP2E
Head development (GO:0060322)
Brain development (GO:0007420)
Neuron fate commitment (GO:0048663)
Regulation of neuron differentiation (GO:0045664)
C
Expressed late-embryonic liver ePrim. enhancer associated TFs
STAT4
CUX1
ID2
FOXP1
SMAD4
IKZF1
NFIB
FOXP2
TCF7L1
SOX13
RUNX1
MEIS1
BCL6
TCF7L2
MEF2C
TEAD4
RREB1
ONECUT2
FOXM1
DNAJC21
NR2F6
ETS2
FOXN2
NFE2L2
DDIT3
SP4
MAFB
HES1
UBP1
KLF7
FOXN3
NFAT5
SP100
Cell fate commitment (GO:0045165)
Transcription coregulator binding, and Activation of the TFAP2 family (STRING cluster: 20714)
Transcriptional regulation by RUNX3 (HSA-8878159)
Known Interactions
from curated databases
experimentally determined
Predicted Interactions
gene neighborhood
gene fusions
gene co-occurrence
Others
textmining
co-expression
protein homology
D
19
1
24
Expressed mECT ePrim. enh. associated TFs
Expressed hNPC ePrim. enh. associated TFs
7
6
163
Expressed mForebrain ePrim. enh. associated TFs
Expressed 7pcw. hBrain ePrim. enh. associated TFs
29
3
55
Expressed mLiver ePrim. enh. associated TFs
Expressed hLiver ePrim. enh. associated TFs

Fig. S12

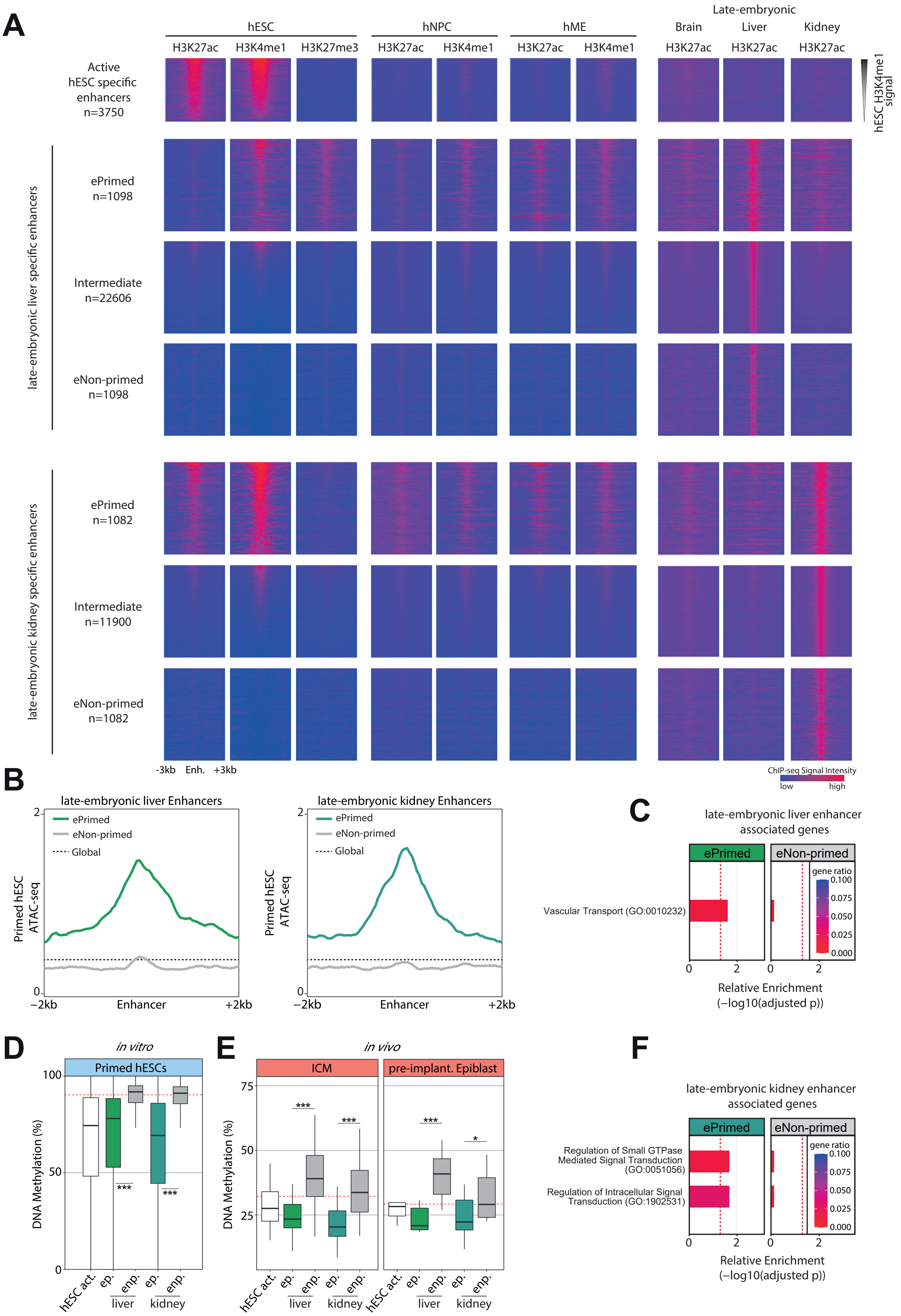

A
hESC
hNPC
hME
Late-embryonic
Brain
Liver
Kidney
H3K27ac
H3K4me1
H3K27me3
Active hESC specific enhancers n=3750
hESC H3K4me1 signal
late-embryonic liver specific enhancers
ePrimed n=1098
Intermediate n=22606
eNon-primed n=1098
late-embryonic kidney specific enhancers
ePrimed n=1082
Intermediate n=11900
eNon-primed n=1082
-3kb
Enh.
+3kb
ChIP-seq Signal Intensity
low
high
B
late-embryonic liver Enhancers
late-embryonic kidney Enhancers
ePrimed
eNon-primed
Global
Primed hESC ATAC-seq
−2kb
Enhancer
+2kb
C
late-embryonic liver enhancer associated genes
ePrimed
eNon-primed
Vascular Transport (GO:0010232)
gene ratio
Relative Enrichment (−log10(adjusted p))
D
in vitro
Primed hESCs
DNA Methylation (%)
hESC act.
ep.
enp.
liver
kidney
E
in vivo
ICM
pre-implant. Epiblast
F
late-embryonic kidney enhancer associated genes
Regulation of Small GTPase Mediated Signal Transduction (GO:0051056)
Regulation of Intracellular Signal Transduction (GO:1902531)

Fig. S13

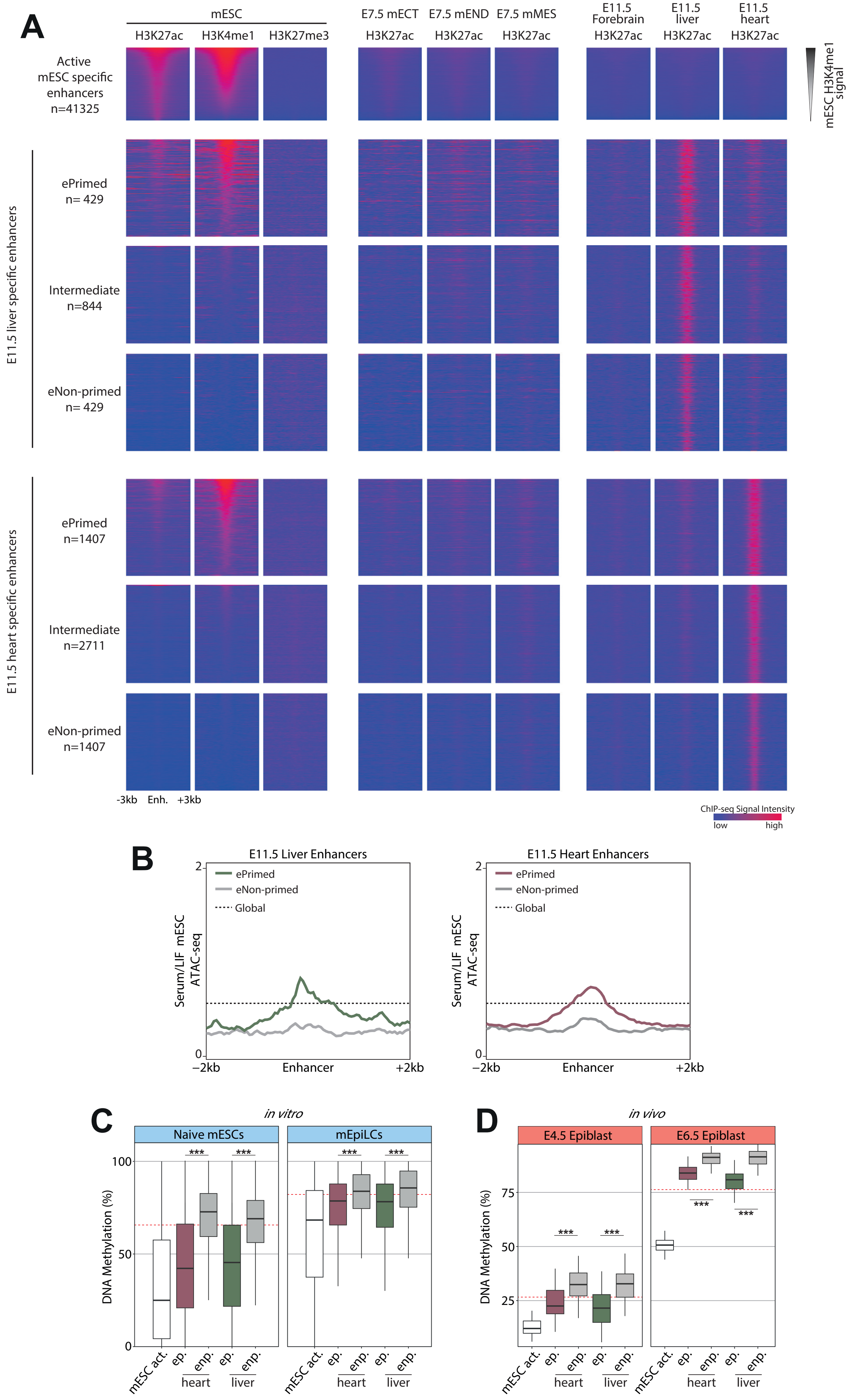
A
mESC
H3K27ac
H3K4me1
H3K27me3
E7.5 mECT
E7.5 mEND
E7.5 mMES
E11.5 Forebrain
E11.5 liver
E11.5 heart
Active mESC specific enhancers n=41325
mESC H3K4me1 signal
E11.5 liver specific enhancers
ePrimed n= 429
Intermediate n=844
eNon-primed n= 429
E11.5 heart specific enhancers
ePrimed n=1407
Intermediate n=2711
eNon-primed n=1407
-3kb
Enh.
+3kb
ChIP-seq Signal Intensity
low
high
B
E11.5 Liver Enhancers
E11.5 Heart Enhancers
ePrimed
eNon-primed
Global
Serum/LIF mESC ATAC-seq
2
0
−2kb
Enhancer
+2kb
C
in vitro
Naive mESCs
mEpiLCs
DNA Methylation (%)
100
50
0
***
mESC act.
ep.
enp.
heart
liver
D
in vivo
E4.5 Epiblast
E6.5 Epiblast
75
25

Fig. S14

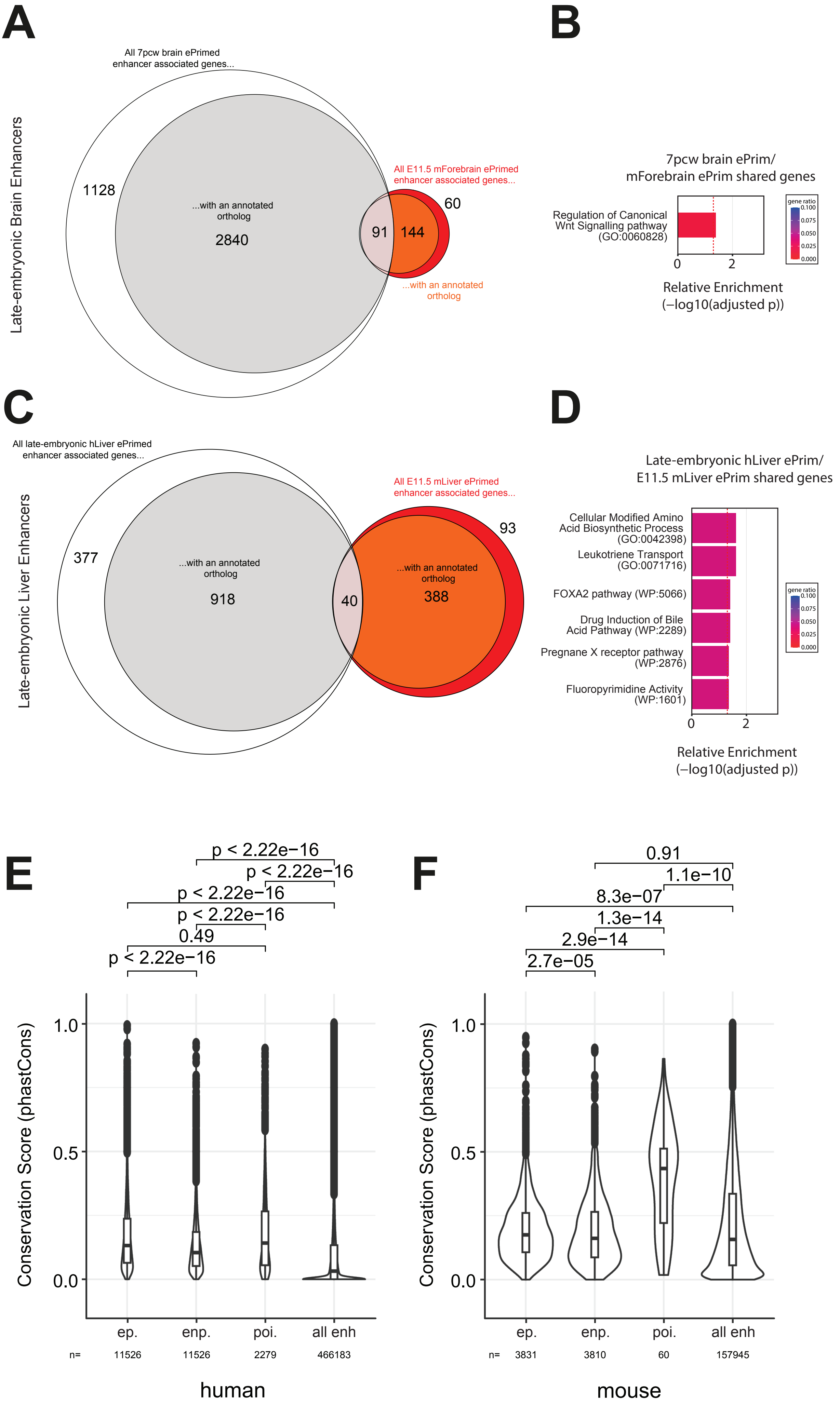
A
B
C
D
E
F
Late-embryonic Brain Enhancers
All 7pcw brain ePrimed enhancer associated genes...
...with an annotated ortholog
1128
2840
91
144
60
All E11.5 mForebrain ePrimed enhancer associated genes...
...with an annotated ortholog
7pcw brain ePrim/ mForebrain ePrim shared genes
Regulation of Canonical Wnt Signalling pathway (GO:0060828)
Relative Enrichment (−log10(adjusted p))
gene ratio
Late-embryonic Liver Enhancers
All late-embryonic hLiver ePrimed enhancer associated genes...
377
918
40
388
93
All E11.5 mLiver ePrimed enhancer associated genes...
Late-embryonic hLiver ePrim/ E11.5 mLiver ePrim shared genes
Cellular Modified Amino Acid Biosynthetic Process (GO:0042398)
Leukotriene Transport (GO:0071716)
FOXA2 pathway (WP:5066)
Drug Induction of Bile Acid Pathway (WP:2289)
Pregnane X receptor pathway (WP:2876)
Fluoropyrimidine Activity (WP:1601)
p < 2.22e−16
0.49
0.91
1.1e−10
8.3e−07
1.3e−14
2.9e−14
2.7e−05
Conservation Score (phastCons)
ep.
enp.
poi.
all enh
n=
11526
11526
2279
466183
3831
3810
60
157945
human
mouse

Fig. S15

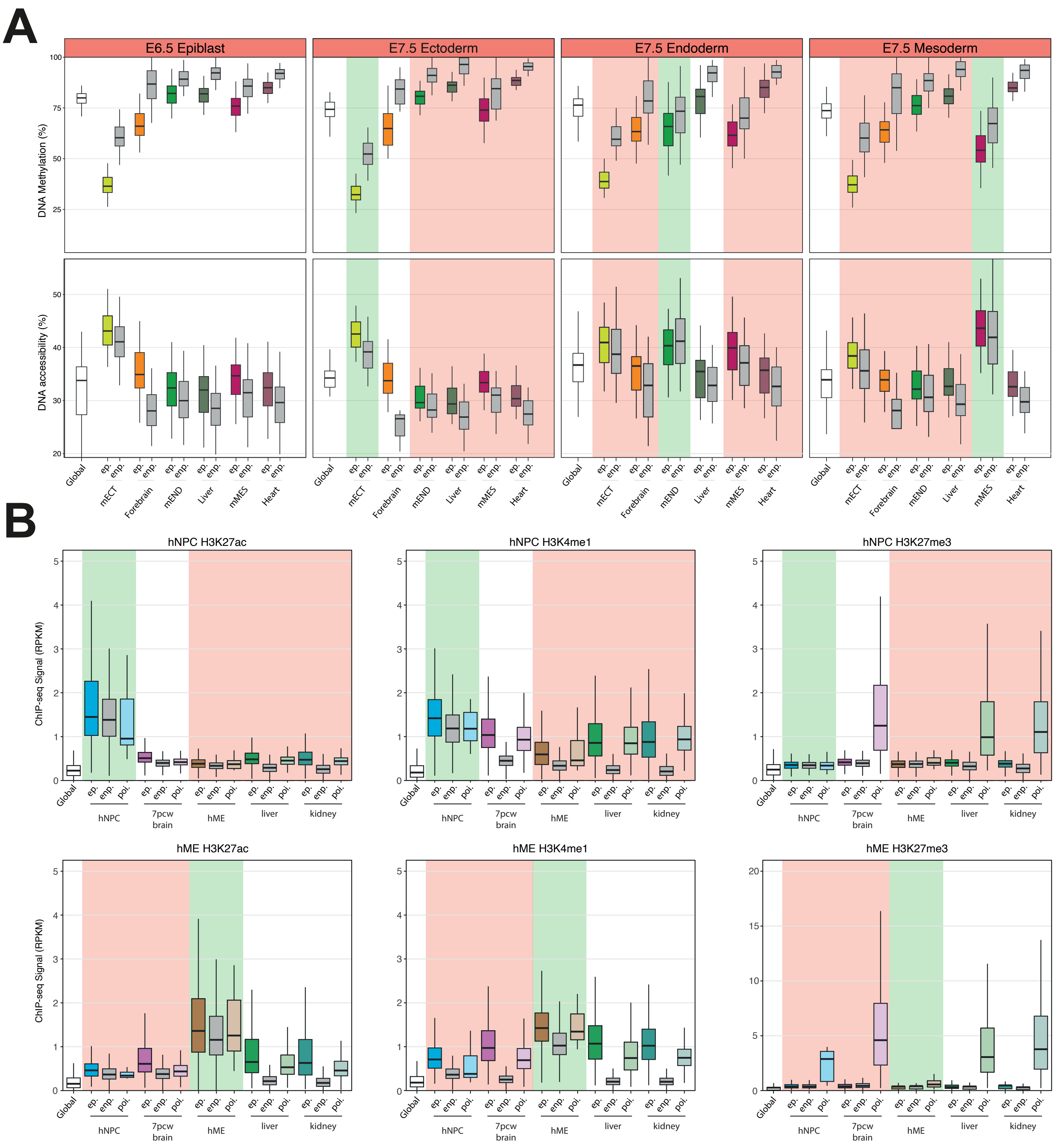
A
E6.5 Epiblast
E7.5 Ectoderm
E7.5 Endoderm
E7.5 Mesoderm
DNA Methylation (%)
DNA accessibility (%)
Global
ep.
enp.
mECT
Forebrain
mEND
Liver
mMES
Heart
B
hNPC H3K27ac
hNPC H3K4me1
hNPC H3K27me3
hME H3K27ac
hME H3K4me1
hME H3K27me3
ChIP-seq Signal (RPKM)
poi.
hNPC
7pcw brain
hME
liver
kidney

Fig. S16

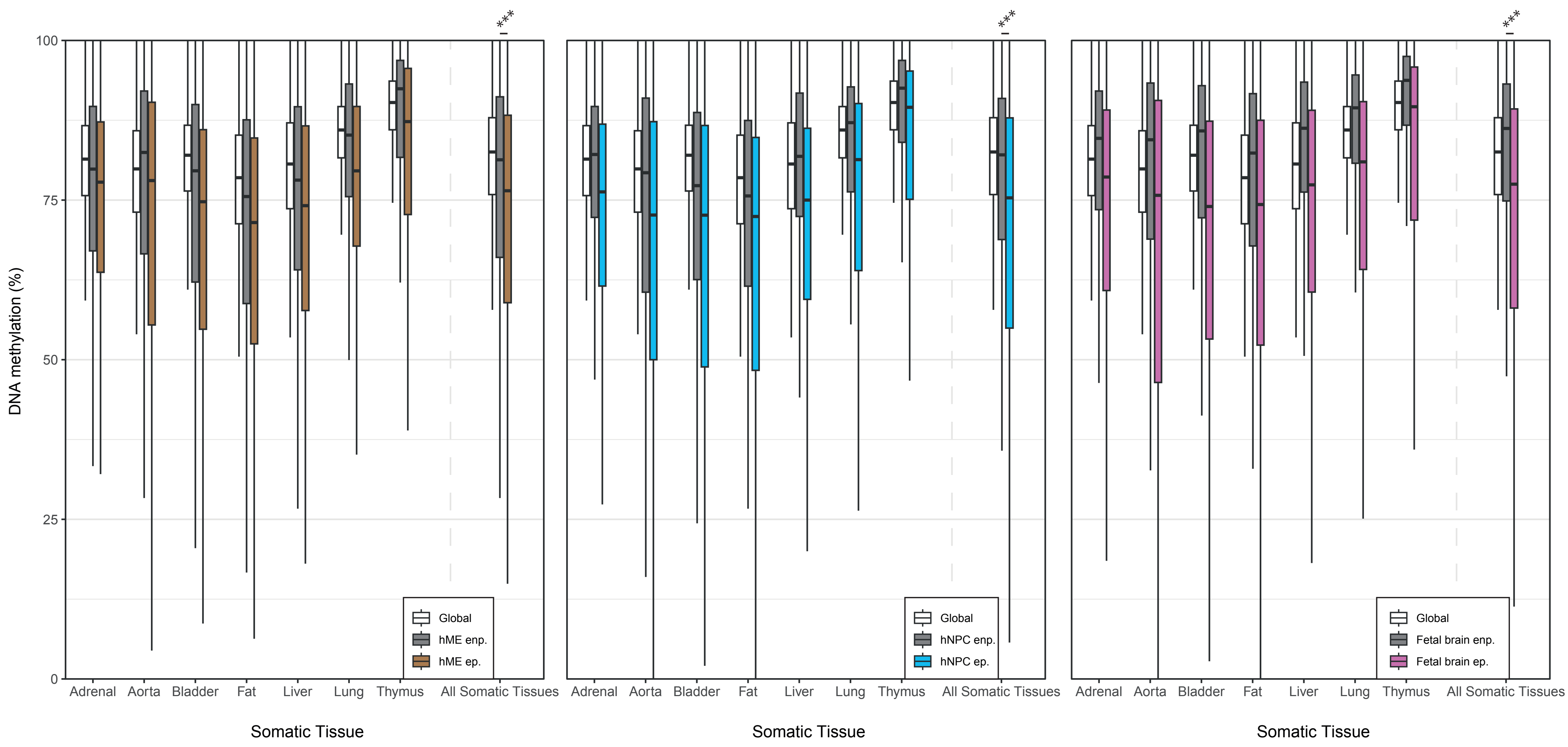

DNA methylation (%)
100
75
50
25
0
***
***
***
Adrenal
Aorta
Bladder
Fat
Liver
Lung
Thymus
All Somatic Tissues
Somatic Tissue
Global
hME enp.
hME ep.
hNPC enp.
hNPC ep.
Fetal brain enp.
Fetal brain ep.

Fig. S17

A

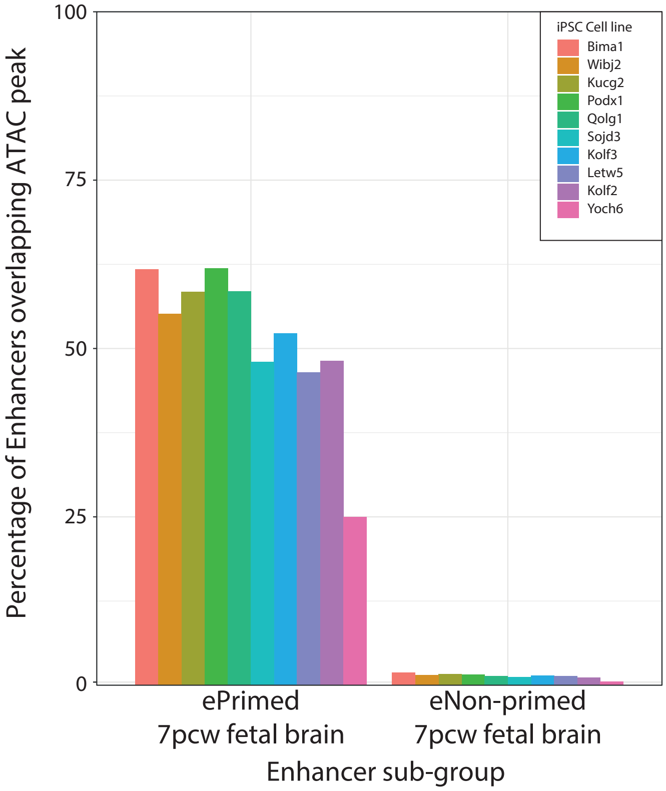

Percentage of Enhancers overlapping ATAC peak
100
75
50
25
0
iPSC Cell line
Bima1
Wibj2
Kucg2
Podx1
Qolg1
Sojd3
Kolf3
Letw5
Kolf2
Yoch6
ePrimed
7pcw fetal brain
eNon-primed
7pcw fetal brain
Enhancer sub-group

B

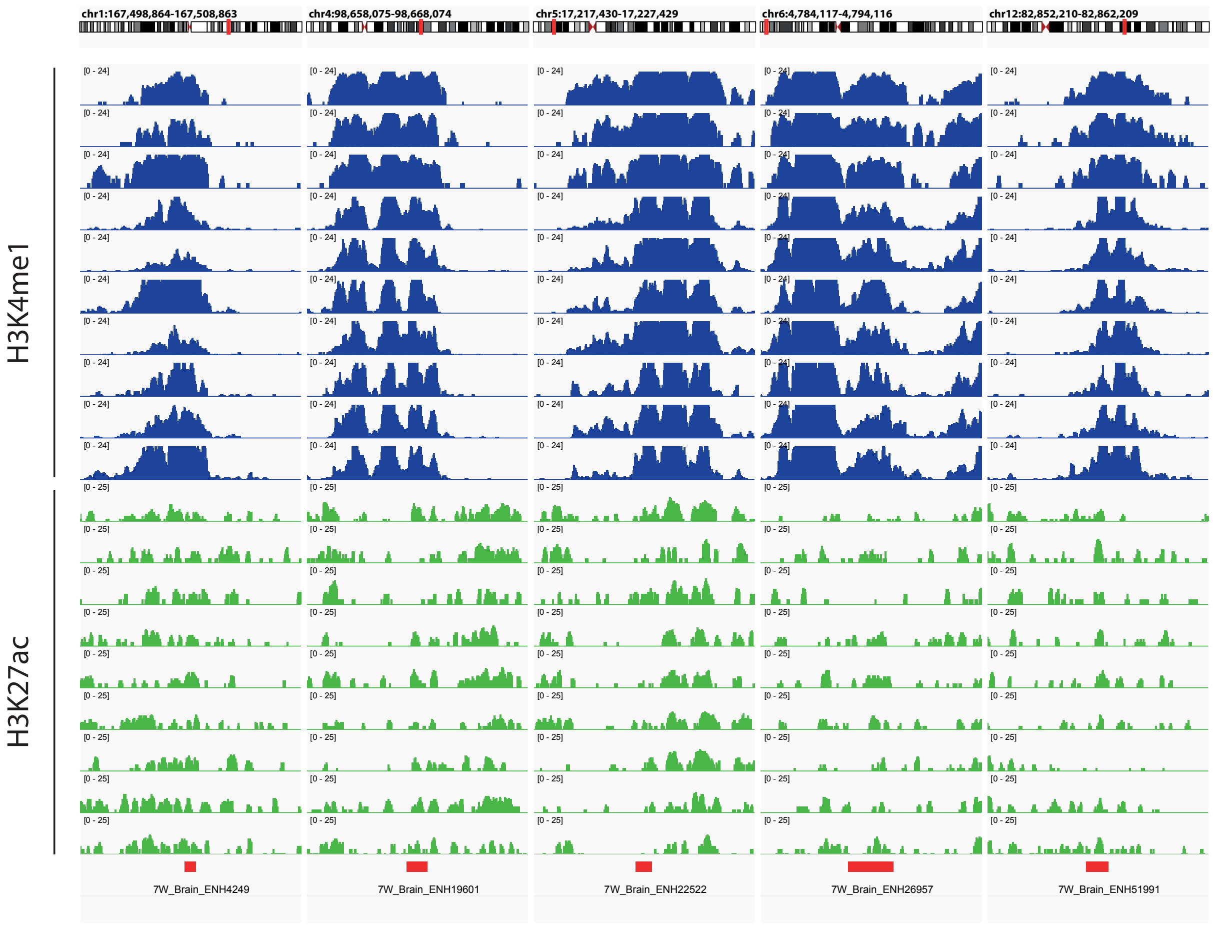

chr1:167,498,864-167,508,863
chr4:98,658,075-98,668,074
chr5:17,217,430-17,227,429
chr6:4,784,117-4,794,116
chr12:82,852,210-82,862,209
H3K4me1
H3K27ac
[0 - 24]
[0 - 25]
7W_Brain_ENH4249
7W_Brain_ENH19601
7W_Brain_ENH22522
7W_Brain_ENH26957
7W_Brain_ENH51991
