# Supplementary material for: Epigenetic priming of mammalian embryonic enhancer elements coordinates developmental gene networks
Source: Genome Biol. 2025 Jul 18;26:214. doi: 10.1186/s13059-025-03658-8 (PMC12272991; doi:10.1186/s13059-025-03658-8)
Supplement: Supplementary file 4 — Additional file 4: Table S3. Accession codes for datasets used. [file 13059_2025_3658_MOESM4_ESM.pdf]

Table S3: Accession codes for datasets used

Organism	Cell type	Data type	Database
Human	hESC	WGBS	GSE75868
	Human somatic tissues	WGBS	GSE16256
	TET/DNMT KO hESC	WGBS	GSE126958
	hESC	ATAC-seq	GSE101074
	iPSC	ATAC-seq	GSE290515
	hESC	ChIP-seq	SRP000941
	hME	ChIP-seq	SRP000941
	hNPC	ChIP-seq	SRP000941
	Human late-embryonic kidney	ChIP-seq	EGAS00001003163
	Human late-embryonic liver	ChIP-seq	EGAS00001003163
	7pcw fetal brain	ChIP-seq	GSE63648
	iPSC	CUT&Tag	GSE290517
	hESC	PCHiC	GSE86821
	hESC	RNA-seq	SRP000941
	hME	RNA-seq	SRP000941
	hNPC	RNA-seq	SRP000941
	Human late-embryonic kidney	RNA-seq	GSE156793
	Human late-embryonic liver	RNA-seq	GSE156793
	Human somatic tissues	RNA-seq	GSE144530
	Human preimplantation embryos	scNMT-seq	GSE279857
Mouse	mESC	WGBS	PRJDB3812
	mEpiLC	WGBS	PRJDB3812
	mESC	ATAC-seq	GSE81679
	mESC	ChIP-seq	GSE223569
	E7.5 mECT	ChIP-seq	GSE125318
	E7.5 mEND	ChIP-seq	GSE125318
	E7.5 mMES	ChIP-seq	GSE125318
	E11.5 forebrain	ChIP-seq	ENCODE: ENCFF001ZRF, ENCFF001ZRD
	E11.5 liver	ChIP-seq	ENCODE: ENCFF001ZRB, ENCFF001ZRL
	E11.5 heart	ChIP-seq	ENCODE: ENCFF001ZRG, ENCFF001ZRE
	mESC	PCHiC	GSE223578
	Mouse preimplantation embryos	COOL-seq	GSE78140
	E4.5/6.5 Epiblast	scNMT-seq	GSE121708
